# Multi-cohort genome-wide association analyses reveal loci underlying circulating liver enzyme levels in African-ancestry populations

**DOI:** 10.21203/rs.3.rs-6941679/v1

**Published:** 2025-07-24

**Authors:** Reagan M. Mogire, Guanjie Chen, Ayo P. Doumatey, Karlijn A. C. Meeks, Mateus H. Gouveia, Jie Zhou, Amy R. Bentley, Daniel Shriner, Adebowale A. Adeyemo, Charles N. Rotimi

**Affiliations:** Center for Research on Genomics and Global Health, National Human Genome Research Institute, Bethesda, MD 20892, USA

**Keywords:** Genome-wide association study, liver enzymes, liver function, African ancestry, alanine aminotransferase

## Abstract

Liver enzymes are critical biomarkers of hepatic metabolism, injury, and systemic homeostasis. Their genetic architecture remains understudied in African-ancestry populations. We addressed this knowledge gap by conducting genome-wide analyses of four liver enzymes in over 55,000 individuals of African ancestry from six cohorts across sub-Saharan Africa, the United States, and the United Kingdom. We identified 31 significant loci, of which 14 were novel, including *TMEM64* and *CRYL1* for alkaline phosphatase, *IMMP2L* for alanine aminotransferase, and *PDE4D* for gamma-glutamyl transferase. Several novel variants exhibited high allele frequencies in African-ancestry populations but were rare or absent in other global populations. Functional annotation indicated that lead variants overlapped liver-active regulatory regions, histone marks, and hepatocyte eQTLs. Colocalization and enrichment analyses implicated pathways related to lipid and carbohydrate metabolism, glycosylation, and immune function. Our findings expand the catalog of genetic variants influencing liver enzymes and advance understanding of the biological mechanisms underlying liver function.

## Background

Liver enzymes are routinely measured biomarkers that reflect hepatic integrity, biliary excretion, and systemic metabolic health. Elevated levels of these enzymes are commonly observed in a range of liver conditions, including metabolic dysfunction-associated steatotic liver disease (MASLD), hepatitis, cholestasis, and hepatocellular carcinoma, as well as in broad range of metabolic disorders such as type 2 diabetes and dyslipidemia^1^.Twin and family-based studies have consistently shown moderate to high heritability for circulating liver enzyme levels–up to 50% for some enzymes^2^. Genetic association studies have identified common variants in genes such as *PNPLA3, GGT1, ABO*, and *ALPL* that influence liver enzyme concentrations and confer risk for liver-related outcomes^3-6^. However, most genetic association studies to date have been conducted in populations of European or East Asian descent^3-6^. Their findings also revealed differences in liver enzyme-associated loci between populations, for example studies in Koreans reveal several differences from European-ancestry GWAS findings ^5,6^.

To date, the genetic architecture of liver enzymes remains understudied in populations of African ancestry, who harbor the most human genetic variation and shorter haplotypes, offering improved resolution for novel variant discovery and fine-mapping^7,8^. Moreover, epidemiological data show that liver enzyme distributions differ by ancestry and ethnicity, with African-ancestry individuals in the United States demonstrating different biochemical profiles and lower prevalence of NAFLD compared to European or Hispanic populations ^9,10^. These differences highlight the need to further investigate the genetics of liver enzymes enzymes in African-ancestry populations through large-scale genomic studies.

To address this gap, we conducted a genome-wide association study (GWAS) of four liver enzymes (alanine aminotransferase [ALT], aspartate aminotransferase [AST], alkaline phosphatase [ALP], and gamma-glutamyl transferase [GGT]) in over 55,000 individuals of African ancestry drawn from six well-characterized cohorts from sub-Saharan Africa, the United States, and the United Kingdom. Our primary goals were to identify novel loci, replicate findings in independent African- and European-ancestry cohorts, and annotate putative regulatory mechanisms using tissue-specific functional genomics data. This study represents the most comprehensive analyses of liver enzyme genetics in African-ancestry populations till date.

## Results

The discovery GWAS meta-analysis included 32,840 participants of African ancestry from five cohorts from sub-Saharan Africa (Nigeria, Ghana, and Kenya), the United States, and the United Kingdom (Supplementary Table 1). Median ages in four of the five cohorts ranged from 42 to 61 years, whereas the fifth cohort (CARDIA, an African American cohort) was by design younger, with a median age of 24.0 (IQR: 21.0, 28.0) years. The proportion of female participants varied from 41% to 72.3% across the cohorts. Overall, the cohorts had relatively high median iody mass index (BMI) values, with the *All of Us* program displaying the highest median BMI at 32.1 (IQR: 27.1, 38.4) and CARDIA the lowest at 24.1 (IQR: 21.5, 28.1). Prevalence of type 2 diabetes (T2D) differed by cohort. The sub-Saharan African AADM study had the highest proportion of T2D cases (48.8%), reflecting its case-control design for T2D, and CARDIA had the lowest proportion (0.9%), likely due to its younger participants. CARDIA also reported the highest frequency of alcohol consumption (80.6%) – Supplementary Table 1.

### Alkaline phosphatase (ALP)

**Fig. 1.**
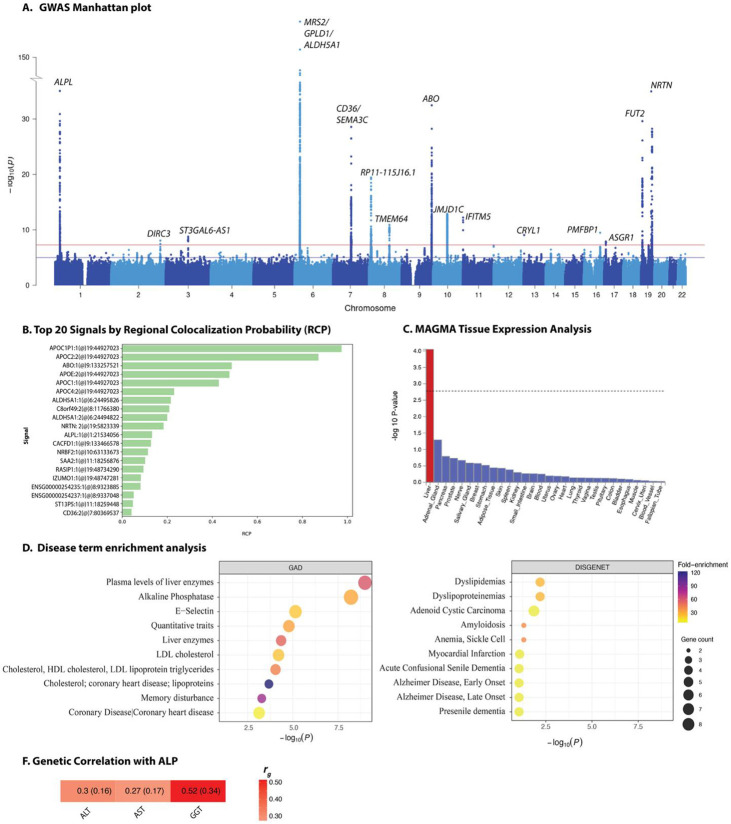
GWAS Manhattan plot for alkaline phosphatase in African-ancestry populations (A), GWAS and eQTL colocalization results using FastENLOC (B), gene-based analyses using MAGMA (C), disease term enrichment analyses in GAD and DISGENET databases (D), and genetic correlation with other liver function enzymes (G). The top 10 enrichment terms are shown. Dot size reflects the number of associated genes, and color indicates fold-enrichment. Heatmap showing pairwise genetic correlations (*r*_g_) among liver biomarkers, values in each cell represent the LDSC point estimate and standard error (SE) in the format *r*_g_ (SE).

The GWAS meta-analysis of all participants of African ancestry in this study identified 17 loci at genome-wide significance (*P* < 5 × 10^−8^) for ALP levels (Table 1, Fig. 1A, and **Supplementary Dataset 1**). Thirteen of the 17 loci have genome-wide significant variants or mapped genes that have been reported in previous GWAS analyses of *ALP* in the GWAS Catalog (Table 1). The remaining four loci were novel, that is, the lead variant, variants in strong linkage disequilibrium (LD) with the lead variant (r^2^ > 0.8), and the mapped gene were neither present in the GWAS Catalog nor near (500 kb apart) a reported locus for ALP (Supplementary Table 2). The novel variants were mapped to the genes Transmembrane Protein 64 *(TMEM64)*, Disrupted in Renal Carcinoma 3 (*DIRC3)*, ST3GAL6 Antisense RNA 1 *(ST3GAL6-AS1)*, and Crystallin Lambda 1 *(CRYL1)* (Table 1, Fig. 1D-I, and Supplementary Fig. 1). We evaluated these novel variants in a separate African-ancestry cohort GWAS (Uganda Genome Resource) and a European-ancestry cohort (UK Biobank). In participants of African ancestry in the Million Veteran Program (MVP), several loci associated with alkaline phosphatase (ALP) were also associated with other phenotypes, including hematological markers such as mean corpuscular hemoglobin concentration, lipid traits like high-density lipoprotein cholesterol and total cholesterol, as well as vitamin B complex deficiencies, anemia, and alanine aminotransferase (ALT) levels (Supplementary Fig. 6) One novel variant, rs140363270, was replicated in the Ugandan cohort, that is, it had the same direction of effect and *P* < 0.05 (β = 0.118, SE = 0.0447, *P* = 0.0084; Supplementary Table 3). Notably, most of the novel variants (*e.g.*, rs144252352, rs140363270, and rs78306989) showed polymorphic allele frequencies in African ancestry populations but were monomorphic or extremely rare in non-African ancestry populations. Statistical fine-mapping produced a 95% credible set of two variants at the *TMEM64* locus and thirteen variants at the *DIRC3* locus, whereas the lead SNPs at *CRYL1* and *ST3GAL6-AS1* each formed singleton credible sets, perhaps reflecting the fine-scale haplotype structure observed in Africans (Supplementary Fig. 2). Across ALP loci with available African-ancestry hepatocyte methylation quantitative trait loci (mQTL) data, *cis*-methylation associations were generally weak and spatially diffuse. At *NRTN*, a locus that has also been previously reported, a few CpG sites reached nominal significance, whereas the *TMEM64* locus showed a sharp cluster of CpGs, most with uniformly positive β estimates, that surpassed the locus-specific Bonferroni threshold, indicating a focal, allele-dependent hyper-methylation hotspot in hepatocytes (Supplementary Fig. 3).

Colocalization of ALP GWAS summary statistics and liver expression quantitative trait locus (eQTL) data revealed the strongest signals in genes previously known to be associated with serum ALP levels such as *ALPL, APOC, ABO*, and *ALDH5A1* (Fig. 1B). Tissue expression association analysis revealed significant enrichment of associated genes in liver tissue (Fig. 1C), consistent with the liver’s role in ALP metabolism. In line with these results, gene-based association analysis identified multiple genes that reached genome-wide significance such as *ALPL, APOC1, CD36, ABO*, and *NRTN* (Supplementary Fig. 4A). To account for the possibility that certain locus-specific associations could be masked in the overall meta-analysis, we conducted cohort-specific GWAS analyses and identified distinct genome-wide significant loci in individual cohorts for ALP, including *KRT18P25* in the AADM cohort and *GPLD1* and *ABO* in the HUFS cohort (Supplementary Fig. 5). In MVP participants of African ancestry , several ALP-associated loci were also associated with other phenotypes, including hematological markers such as mean corpuscular hemoglobin concentration, lipid traits like high-density lipoprotein cholesterol and total cholesterol, as well as vitamin B complex deficiencies, anemia, and ALT levels (Supplementary Fig. 6)

The top gene-set enrichment analysis terms included plasma liver enzyme levels—particularly ALP itself—alongside dyslipidaemia, quantitative lipid traits (HDL cholesterol, LDL cholesterol, and triglycerides), and downstream cardiometabolic outcomes such as coronary heart disease (Fig. 1D). The associated gene set was also significantly enriched for glycosyltransferase activity, blood group antigen biosynthesis, and serine protease homolog domains. The leading biological process categories focused on lipid metabolism, lipid transport, and receptor-mediated endocytosis (Supplementary Fig. 7A). These findings suggest that ALP-associated variants mark genes and pathways that are involved in hepatic function and lipid handling.

The *TMEM64* locus for ALP (lead variant rs78306989) harbors multiple SNPs with high Combined Annotation Dependent Depletion (CADD) scores, indicating potential deleterious effects. Additionally, these SNPs are located within regions marked as active promoters or enhancers in liver tissue, suggesting potential regulatory activity (Supplementary Fig. 7A). *TMEM64* exhibits moderate expression in the liver in comparison to other tissues (Supplementary Fig. 8 and **Supplementary Dataset 3**). Gene perturbation analysis revealed that *TMEM64* expression is increased upon hepatocyte nuclear factor 4 alpha (HNF4α) depletion in HepG2 hepatocellular carcinoma cells (Supplementary Fig. 8). In contrast, its expression is downregulated in response to *FOXO1* knockout in mouse T regulatory cells, GK knockout in mouse brown adipose tissue, *HNF4A* knockdown in HepG2 cells, and Zfp36l2 deficiency in fetal mouse liver at embryonic day 14.5 (E14.5), suggesting a potential regulatory role for *TMEM64* in liver development and metabolic processes (Supplementary Fig. 8 and comprehensive results in **Supplementary Dataset 3**). The CTD Gene–Disease Associations dataset shows that *TMEM64* is associated with liver diseases (standardized value, SV = 1.45), drug-induced liver injury (SV = 1.59), fatty liver (SV = 1.04), liver neoplasms (SV = 1.34), hepatocellular carcinoma (SV = 1.27), and acute liver failure (SV = 1.03), suggesting its potential involvement in hepatic pathology. Additional associations with metabolic disorders such as hyperglycemia (SV = 1.32), insulin resistance (SV = 1.20), and hypertriglyceridemia (SV = 1.13) further implicate *TMEM64* in glucose and lipid metabolism (**Supplementary Dataset 3**).

### Alanine transaminase

**Fig. 2.**
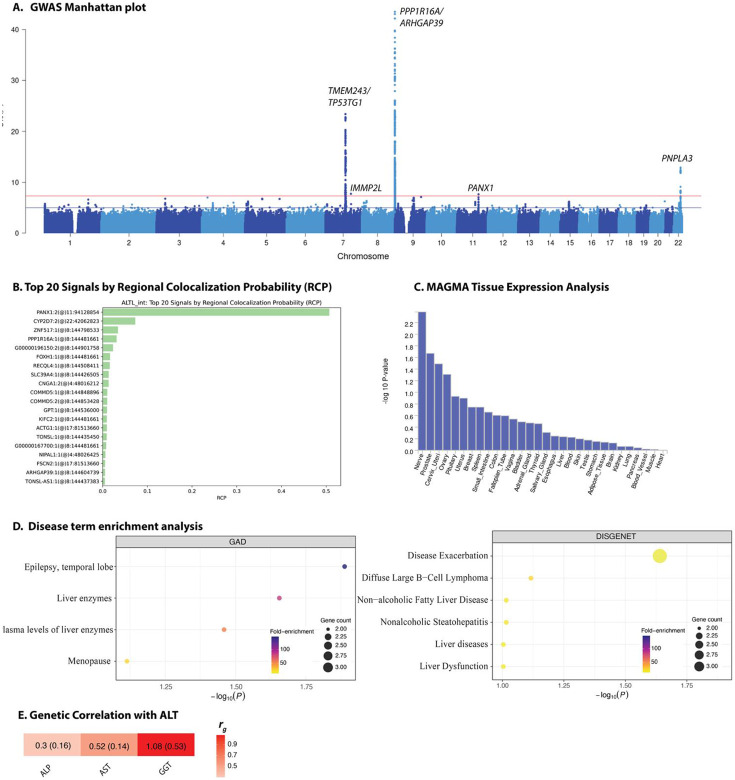
GWAS Manhattan plot for alanine aminotransferase in African-ancestry populations (A), GWAS and eQTL colocalization results using FastENLOC (B), gene-based analyses using MAGMA (C), disease term enrichment analyses in GAD and DISGENET databases (D), and genetic correlation with other liver function enzymes (E). The top 10 enrichment terms are shown. Dot size reflects the number of associated genes, and color indicates fold-enrichment. Heatmap showing pairwise genetic correlations (*r*_g_) among liver biomarkers, values in each cell represent the LDSC point estimate and standard error (SE) in the format *r*_g_ (SE).

Five loci reached genome-wide significance in the meta-analysis GWAS for ALT levels (Fig. 2A, **Supplementary Dataset 2**, Table 1, and Fig. 2D-**I**). Except for the novel intronic variant rs138797771 (located within *IMMP2L*), all the genome-wide significant variants have been previously reported in GWAS of ALT (Table 1 and Supplementary Table 2). Both rs137964419 and rs138797771 are monomorphic or rare outside non-African populations (1000 Genomes). Replication analyses in African-ancestry participants from the MVP showed consistent associations for all lead variants except the novel variant rs138797771. Notably, rs137964419 (*KIAA1324L)*, rs4753126 (*PANX1*), and rs738408 (*PNPLA3*) were also associated with aspartate aminotransferase (AST) levels in the same PheWAS, suggesting that some ALT loci may exhibit pleiotropic effects across liver enzyme traits (Supplementary Fig. 10). The SNP rs738408 (*PNPLA3*) was also associated with chronic liver diseases, abnormal function of the liver, and other non-alcoholic liver disease, highlighting the clinical relevance of the locus.

Annotation of the five ALT-associated loci point to regulatory mechanisms, some of which converge on hepatic biology. The common intronic variant rs2721150 in *PPP1R16A* shows robust liver and HepG2 enhancer signatures, gains RREB1/YY1 binding, and acts as a multi-tissue eQTL, supporting a broad transcriptional effect that includes hepatocytes. The promoter-proximal variant rs4753126 (upstream of *PANX1*) sits in a universally active TSS region, is bound by POL2, STAT3, ELF1, and drives strong eQTL signals in blood and thyroid, pointing to a systemic inflammatory axis that could secondarily modulate liver enzymes. Finally, the extensively replicated synonymous variant rs738408 in *PNPLA3*—a gene central to NAFLD pathogenesis— is located within a highly conserved, epigenetically active hepatic region and alters COMP1/other transcription factor motifs, providing a molecular link between genetic variation, hepatic lipid metabolism, and elevated ALT. There was evidence of *cis*-mQTL signals at the *PANX1* locus, with several CpG sites showing nominally significant positive effect associations (Supplementary Fig. 3). The SNP rs137964419 lies within an H3K4me1-marked enhancer that harbors CTCF/cohesin and RXRA motifs, consistent with a role in long-range chromatin looping. On the other hand, rs138797771 sits in an intron of *IMMP2L* and weakens conserved TAL1 and GATA motifs. However, the absence of liver-specific histone marks suggests any impact on ALT via this mechanism is indirect or tissue-restricted.

Colocalization analysis with liver eQTL data indicated strong regional colocalization for *PANX1*, and substantial evidence of colocalization was also observed for *CYP2D7* and *PPP1R16A* (Fig. 2B). Tissue expression analysis revealed nominal signals for the fallopian tube, uterus, spleen, and liver (Fig. 2B), though none reached statistical significance. Gene-based association analysis highlighted previously known genes such as *PNPLA3* and *ABCB1, CROT*, and *PPP1R16A* (Supplementary Fig. 4B). Several variants reached genome-wide significance in cohort-specific GWAS for African-ancestry participants in the UK Biobank and the *All of Us* Research Program (Supplementary Fig. 11). Gene-set enrichment analysis top disease terms clustered around liver injury and fatty liver phenotypes, including general “liver dysfunction/liver diseases,” non-alcoholic fatty liver disease (NAFLD), and steatohepatitis, alongside expected associations with liver enzyme traits themselves (Fig. 2D). At the functional level, this gene set was significantly enriched for DNA-binding activity and the broad biological process of intracellular transport (Supplementary Fig. 7B), suggesting potential roles in transcriptional regulation and subcellular trafficking within hepatocytes.

### Aspartate transaminase

**Fig. 3.**
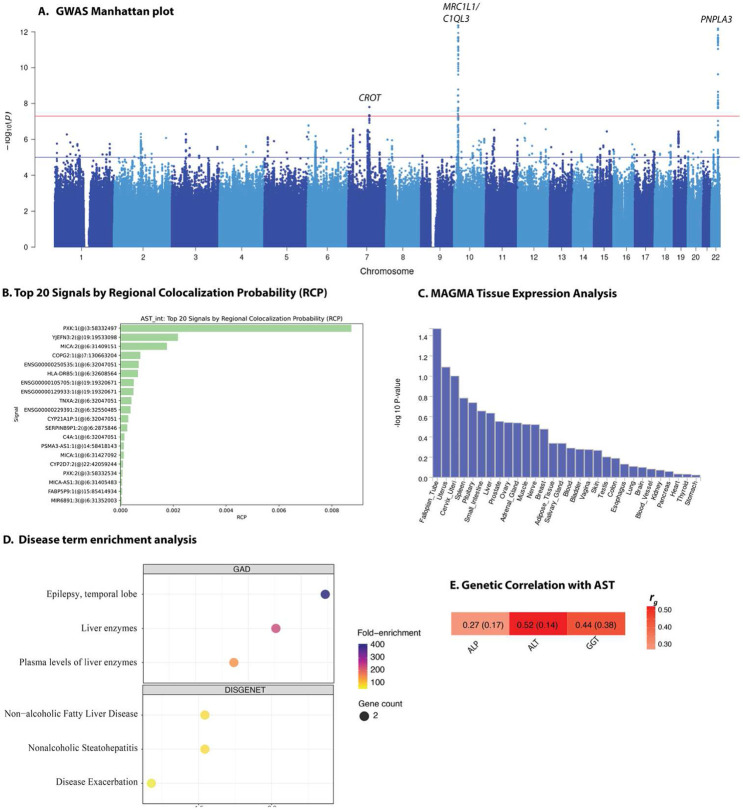
GWAS Manhattan plot for aspartate aminotransferase in African-ancestry populations (A), GWAS and eQTL colocalization results using FastENLOC (B), gene-based analyses using MAGMA (C), disease term enrichment analyses in GAD and DISGENET databases (D), and genetic correlation with other liver function enzymes (E). The top 10 enrichment terms are shown. Dot size reflects the number of associated genes, and color indicates fold-enrichment. Heatmap showing pairwise genetic correlations (*r*_g_) among liver biomarkers, values in each cell represent the LDSC point estimate and standard error (SE) in the format *r*_g_ (SE).

The meta-analysis GWAS of AST levels revealed five loci at genome-wide significance (Fig. 3A and **Supplementary Dataset 1**). The significant variants in these loci, variants in strong LD with the lead SNP, or the corresponding mapped gene have been reported in previous GWAS analyses except for two novel signals—rs7086539 (*C1QL3*) and rs56331215 (*MRC1L1*) (**Supplementary Dataset 2**, Table 1, Fig. 3D-E, Supplementary Fig. 1, and Supplementary Table 2). 10 out of 13 mapped genes overlapped with ALT-associated genes (Supplementary Fig. 1). Association at the *C1QL3* variant rs7086539 was replicated in the Ugandan cohort but rs56331215 (*MRC1L1*) was missing in all replication cohorts (Supplementary Table 3).

Gene-set enrichment analysis of AST-associated loci revealed statistically significant associations with liver enzyme levels, and temporal-lobe epilepsy (Fig. 3D), as well as with non-alcoholic fatty liver disease (NAFLD), its progressive form, non-alcoholic steatohepatitis (NASH), and a broad term indicating liver disease exacerbation. For Gene Ontology (GO) molecular function terms, two categories emerged as significantly enriched: translocase activity and acyltransferase activity (Supplementary Fig. 7C). These functions implicate proteins involved in the transport and remodeling of lipids across intracellular membranes, suggesting a potential mechanistic link between AST regulation and intracellular lipid handling.

Functional annotation of the associated loci indicates their potential involvement in hepatic pathways. The *C1QL3* variant rs7086539 alters IK-3 and RFX5 binding motifs, implying possible transcriptional modulation. Colocalization with liver eQTL data pointed to strong signals in *PXK, YJEFN3*, and *MICA* (Fig. 3B), while tissue expression analysis revealed nominal associations in nerve, prostate, and cervix, though none reached genome-wide significance (Fig. 3C). Gene-based analyses identified several genes — *PNPLA3, MRC1L1, MRC1, TMEM236*, and *CROT* —at genome-wide significance (Supplementary Fig. 4C). In the African-ancestry subset of the UK Biobank, a single significant locus in *RYR3* was detected (Supplementary Fig. 12). For both the *C1QL3* and *MRC1L1* novel loci, statistical fine mapping identified a single variant in the credible set (Supplementary Fig. 2). No *cis*-mQTLs were detected at the AST-associated loci in hepatocytes (Supplementary Fig. 3). The variants rs576738951 and rs12485100 were associated with both ALT and AST levels in African ancestry participants in the MVP (Supplementary Fig. 13)

### Gamma-glutamyl transferase

**Fig. 4.**
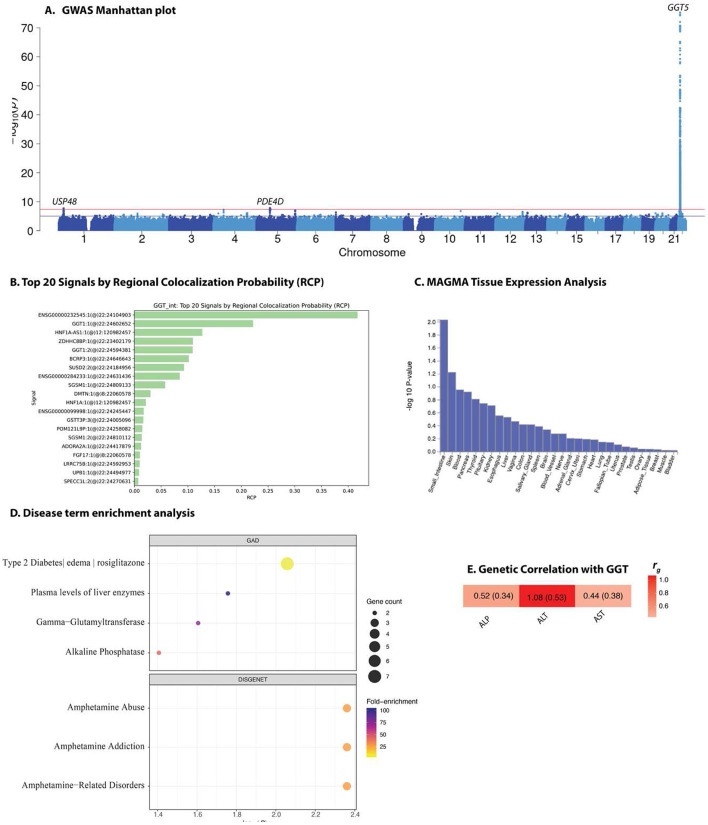
GWAS Manhattan plot for gamma-glutamyl transferase in African-ancestry populations (A), GWAS and eQTL colocalization results using FastENLOC (B), gene-based analyses using MAGMA (C), disease term enrichment analyses in GAD and DISGENET databases (D), and genetic correlation with other liver function enzymes (E). The top 10 enrichment terms are shown. Dot size reflects the number of associated genes, and color indicates fold-enrichment. Heatmap showing pairwise genetic correlations (*r*g) among liver biomarkers, values in each cell represent the LDSC point estimate and standard error (SE) in the format *r*_g_ (SE).

The overall meta-analysis for GGT levels showed four loci at genome-wide significance (Fig. 4A, **Supplementary Dataset 1**, and **Supplementary Dataset 2**). Many variants in the *GGT5* and *USP48* loci have been reported in previous GGT GWAS analyses, while associations at the *PDE4D* and *MYO18B* loci are novel (Table 1 and Supplementary Table 2). Colocalization analyses with liver eQTL data indicated strong signals in *GGT1, HNF1A-AS1*, and *ZDHHC8BP* (Fig. 4B). Tissue expression analysis showed nominal enrichment in the small intestine, skin, and blood (Fig. 4C). Gene-based association analysis identified 16 genome-wide significant genes, including *USP48, PDE4D, HNF1A, SGSM1*, and *GGT5* (Supplementary Fig. 4C). In cohort-specific GWAS, the same genome-wide significant locus on chromosome 22 (*GGT5)* was confirmed across all studies (Supplementary Fig. 14). The variant rs6675677 was associated with white blood cell count (WBC), neutrophil and monocyte counts, as well as body mass index (BMI), in MVP African ancestry participants (Supplementary Fig. 15). There was little evidence of replication of the novel loci in Ugandans and Europeans (Supplementary Table 3). The fine-mapping credible set at the *PDE4D* locus contained two SNPs including the lead SNP, whereas at *MYO18B*, the lead SNP was the only variant in the credible set (Supplementary Fig. 2). No *cis*-mQTLs were detected at the GGT-associated loci in hepatocytes (Supplementary Fig. 3).

Functional annotation suggests a transcriptional mechanism of these loci. rs6675677 is a strong *cis*-eQTL for *USP48* in thyroid tissue (*P* ≈ 1.29 × 10^−25^). Since *USP48* deubiquinates components of the NF-κB pathway and DNA damage machinery, altered expression could indirectly influence the hepatocellular stress responses that raise GGT levels. The SNP rs333169—a variant with r^2^ > 0.8 with rs6675677—lies on a pronounced H3K4me1 enhancer peak in HepG2 cells, situating the haplotype within liver-active chromatin. Further downstream, the *GGT5* variant rs199882393–an insertion predicted to enhance FOXA2/Fox-family binding–may boost enhancer activity within a genomic region involved in glutathione metabolism, consistent with FOXA2’s role in opening chromatin at hepatic metabolic genes^11^.

Gene-set enrichment analysis of GGT-associated loci revealed a distinctive signature characterized by antioxidant and xenobiotic-response pathways. Using the GAD database, the strongest enrichment signal corresponded to a composite term encompassing type 2 diabetes, oedema, and rosiglitazone response. This was followed by associations with general plasma liver enzyme traits, as well as specific enzymes such as γ-glutamyl transferase and alkaline phosphatase (Fig. 1D). In the DISGENET resource, all associations related to disorders involving amphetamine abuse or addiction. These suggest that genetic variants influencing GGT levels may intersect with drug-response pathways for both thiazolidinediones and psychostimulants, alongside their established role in regulating hepatic enzyme activity. For Gene Ontology (GO) terms, enrichment was observed in two molecular function categories—hydrolase activity and protease activity—reflecting the known enzymatic functions of GGT (Supplementary Fig. 17D). On the biological process, the only significantly enriched term was glutathione biosynthesis, highlighting the enzyme’s canonical involvement in the γ-glutamyl cycle and antioxidant defense.

### Genetic correlation and heritability

SNP-based narrow-sense heritability estimates and genetic correlation for the four liver enzymes were calculated using LD Score Regression (LDSC)^12^, a method that distinguishes true polygenic signal from confounding factors in GWAS summary statistics. SNP-based narrow-sense heritability estimates for the four liver enzymes studied ranged from 6.1% to 18.6%. The highest heritability was observed for ALP (*h^2^* = 0.186, SE = 0.038), followed by ALT (*h^2^* = 0.096, SE = 0.026), GGT (*h^2^* = 0.074, SE = 0.067), and AST (*h^2^* = 0.061, SE = 0.022). LDSC intercepts were close to 1 across all traits, ranging from 1.008 to 1.035, indicating minimal confounding due to population stratification or cryptic relatedness. LDSC analysis of pairwise genetic correlation to assess shared genetic architecture among the liver enzyme traits, revealed evidence of moderate to strong genetic overlap across the liver enzymes. The genetic correlation between ALT and AST was strongest and statistically significant (r_g_ = 0.52, SE = 0.14, *P* = 0.0003). We also observed a strong genetic correlation (r_g_ = 1.08, SE = 0.53, *P* = 0.042) between ALT and GGT (note: the point estimate slightly exceeds the theoretical upper bound of 1, which is a known artefact of LDSC under conditions of imprecise estimation due to large standard errors or modest heritability). A moderate positive genetic correlation was observed between ALP and ALT (r_g_ = 0.30, SE = 0.16, *P* = 0.053), suggesting the evidence of partially shared genetic influences (Fig. 1). Similarly, demonstrated a positive correlation ALP and AST (r_g_ = 0.27, SE = 0.17, *P* = 0.098), ALP and GGT (r_g_ = 0.52, SE = 0.34, *P* = 0.13) and AST and GGT (r_g_ = 0.44, SE = 0.38, *P* = 0.25), although these associations did not reach statistical significance.

## Discussion

This genome-wide analysis has substantially expanded the scale and scope of genetic studies of liver enzymes in African-ancestry populations. By integrating data from cohorts in sub-Saharan Africa and from national biobank-scale cohorts in the United States and the United Kingdom, we successfully identified 31 genome-wide significant loci across the four enzymes (including 17 for ALP, five each for ALT and AST, and four for GGT). Several loci represent novel associations, of which we replicated a subset in an independent African-ancestry cohort. We observed a substantial genetic correlation among the liver enzymes (especially high between ALT and AST), suggesting that shared regulatory and/or metabolic pathways underly the genetic variation in these biomarkers.

Our GWAS of ALP revealed 17 genomic loci, 13 of which have been reported in previous GWAS analyses, alongside four novel loci. While some variants replicated in European-ancestry cohorts indicating trans-ancestry relevance, other variants (*e.g.*, rs144252352 [*DIRC3*], rs140363270 [*ST3GAL6-AS1*], and rs78306989 [*TMEM64*]) have polymorphic allele frequencies only in African-ancestry populations, reinforcing the importance of capturing the full range of human variation in GWAS and other genomic studies. The discovery that some of these variants (*e.g.*, rs34182948 [*NRTN*] and rs112528434 [*APOC1*]) also display pleiotropic associations with other metabolic traits—including vitamin B complex deficiencies and triglyceride and total cholesterol levels—further supports the idea that liver enzyme pathways are integral to broader metabolic homeostasis ^13^. *TMEM64* emerged as a locus associated with ALP levels in populations of African ancestry, with functional evidence indicating a role in liver function and metabolic regulation.

Five genome-wide significant loci were identified for ALT, including a novel variant in *IMMP2L*. These loci have strong functional and regulatory evidence supporting their involvement in hepatic processes. Notably, replication of nearly all lead variants (except for rs138797771) in an independent African-ancestry cohort from the Million Veteran Program underscores the robustness of our findings. The shared associations of several loci with AST in phenome-wide analyses further indicate pleiotropic regulatory mechanisms that span liver enzyme phenotypes as previously described^14^. We identified two novel signals —rs7086539 (*C1QL3*) and rs56331215 (*MRC1L1*) —for AST levels with plausible roles for these variants in hepatic gene regulation. The SNP rs7086539 disrupts IK-3 and RFX5 transcription factor motifs near *C1QL3*, implicating potential immune and transcriptional modulation relevant to hepatic function. Meanwhile, *CROT* has been linked to lipid metabolism and mitochondrial fatty acid oxidation. *GGT5* and *USP48* have been consistently implicated in prior GGT GWASs ^13^, while *PDE4D* and *MYO18B* represent novel loci. However, these novel signals did not replicate in either the African (Ugandan) or European-ancestry cohorts, possibly due to differences in LD patterns and/or other issues that influence replication across populations. Nonetheless, it should be noted that colocalization analyses with liver eQTL data and functional annotation support their roles in liver biology.

Across all four liver enzymes, a set of convergent themes emerged that underscore a partially shared allelic architecture suggestive of coordinated regulation across hepatocellular integrity, lipid homeostasis, and antioxidant defense. Mechanistically, both novel and established loci mapped to genes involved in three interrelated biological pathways: (i) hepatic lipid metabolism and lipoprotein remodeling (e.g., *TMEM64, PNPLA3, CD36, CROT*); (ii) oxidative stress and xenobiotic detoxification, particularly via the glutathione and γ-glutamyl cycles (e.g., *GGT5, USP48, PDE4D*); and (iii) glycosyl- and phosphotransferase-mediated post-translational modification, which links ALP and GGT activity to receptor recycling and endocrine regulation. These pathways are concordant with functional enrichment signals for glycosyltransferase, hydrolase, and lipid transport activity, providing molecular support for the observed epidemiological association between liver enzymes and cardiometabolic traits^15,16^.

This study has several strengths. Foremost among them is the scale and breadth of the analysis, representing one of the largest genome-wide association studies of liver enzyme traits conducted in African-ancestry populations to date, with participants drawn from sub-Saharan Africa, the United States, and the United Kingdom. This broad representation includes individuals with shared genetic ancestry but diverse environmental exposures, offering a unique opportunity to disentangle genetic effects from environmental context. As a result, the study captures substantial genetic and environmental heterogeneity and enhances the generalizability of the findings within African-ancestry populations Notably, several lead variants are common or only polymorphic in African ancestry populations yet rare or absent in other groups, highlighting how ancestrally diverse datasets can uncover genetic loci and regulatory elements that may remain undetected in single ancestry studies^8^ . Second, the use of high-resolution genome-wide data enabled robust detection of genetic associations, including the identification of novel loci. Nonetheless, the sample size remains modest compared to large-scale GWAS conducted in European and East Asian populations, potentially limiting statistical power to detect variants with smaller effect sizes^17^. Additionally, in common with most meta-analysis studies, heterogeneity in study design, environmental exposures, and dietary patterns across cohorts may introduce residual confounding, despite the use of meta-analytic methods to account for between-study variability

In summary, this meta-analysis study of four key liver function enzymes in African-ancestry populations has expanded the repertoire of genetic loci influencing serum enzyme levels. The findings point to biological pathways central to liver physiology and the regulatory relevance of several candidate genes to liver enzyme variation and liver function. Similar studies will be critical for successfully capturing the full range of human variation for liver function enzymes and for ensuring that precision medicine strategies are informed by and applicable to individuals across all populations.

## Methods

### Ethics statement

All contributing studies obtained approval from their respective Institutional Review Boards or Ethics Committees, and all participants provided written, informed consent at the time of enrollment, including consent for genetic analyses. The present analysis was conducted under protocols approved by the Institutional Review Boards of the respective institutions managing each dataset.

### Study cohorts

This study involved analysis of data from six cohorts comprising individuals of African ancestry: (1) the Africa America Diabetes Mellitus (AADM) study, (2) the Howard University Family Study (HUFS), (3) the Coronary Artery Risk Development in Young Adults (CARDIA) study, (4) the Multi-Ethnic Study of Atherosclerosis (MESA), (5) African-ancestry participants from the UK Biobank, and (6) African American participants enrolled in the All of Us Research Program. A brief overview of each cohort is provided below, with demographic and clinical characteristics presented in Supplementary Table 1.

The *Africa America Diabetes Mellitus (AADM)* study is a collaborative research initiative designed to investigate the genetic and environmental determinants of type 2 diabetes in African populations, including participants from Ghana, Nigeria, and Kenya. By combining comprehensive phenotypic data with dense genotyping, AADM provides critical insights into the genetic architecture of cardio-metabolic traits in African populations^40-44^.

The *Howard University Family Study (HUFS)* is a population-based cohort focused on African Americans residing in the Washington, D.C. metropolitan area. Utilizing a family-based recruitment design, HUFS aims to elucidate the genetic underpinnings of complex diseases such as hypertension and obesity, and collects extensive information on lifestyle, psychosocial variables, and anthropometry^45^.

The *Coronary Artery Risk Development in Young Adults (CARDIA)* study is a multi-center, longitudinal study that follows a racially and geographically diverse cohort of adults aged 18–30 at baseline. CARDIA tracks cardiovascular and metabolic health across the life course and includes a substantial subset of African American participants^46^. Data for the CARDIA study were accessed through dbGaP (phs000285)

The *Multi-Ethnic Study of Atherosclerosis (MESA)* was designed to study the progression of subclinical cardiovascular disease in a multiethnic cohort aged 45–84 years. MESA integrates high-resolution imaging, clinical, and genomic data to assess early atherosclerotic changes and their determinants, and includes a substantial representation of African Americans^47^. Data for MESA was accessed through dbGaP (phs000209).

The *UK Biobank* is a large-scale, population-based cohort comprising approximately 500,000 participants aged 40–69 at enrollment, primarily of European ancestry but also including individuals of African and Afro-Caribbean descent. Rich phenotypic data, genome-wide genotyping, and linkage to electronic health records support genomic investigations^48^.

The *All of Us Research Program* is a national precision medicine initiative in the United States that aims to enroll over one million participants, with a strong emphasis on underrepresented minorities. The program collects genomic, clinical, and environmental data to explore how genetic and non-genetic factors contribute to health disparities, and includes substantial African American representation^49^.

### Phenotype Assessment

Across cohorts, participants underwent clinical assessments that included anthropometric measurements (*e.g.*, height and weight) and venous blood collection for biochemical assays. Four liver enzymes — alanine aminotransferase (ALT), aspartate aminotransferase (AST), alkaline phosphatase (ALP), and gamma-glutamyl transferase (GGT) — were measured using standardized automated protocols at each study site. Individuals with missing phenotype data, outlier or implausible enzyme levels, or insufficient covariate information were excluded from downstream analyses. When applicable, covariates included age, sex, body mass index (BMI), alcohol consumption, and type 2 diabetes case/control status.

### Genotyping and Imputation

Participants in the AADM study were genotyped on high-density GWAS arrays (either the Affymetrix^®^ Axiom^®^ Genome-Wide PanAFR Array Set or the Illumina Multi-Ethnic Genotyping Array). Other cohorts followed protocols detailed in Supplementary Table 1. Technical quality control (QC) included removing samples with individual call rates ≤95%, SNP call rates ≤95%, Hardy–Weinberg equilibrium (HWE) *P* < 1 × 10^−6^, and minor allele frequency (MAF) <0.01. For the UK Biobank and *All of Us* Research Program cohorts, which utilized precomputed association statistics, genotyping and QC procedures were conducted independently according to their respective study protocols. After QC, genotypes were phased and imputed via the TOPMed Imputation Server (https://imputation.biodatacatalyst.nhlbi.nih.gov/). Post-imputation variants were filtered based on an INFO score ≥0.3 and MAF ≥0.001.

### Genome-Wide Association Study (GWAS) Analysis

We conducted genome-wide association analyses for each liver enzyme using the SAIGE (v0.44.6) software package, which applies a generalized linear mixed model (GLMM) framework optimized for related samples. Prior to analysis, enzyme levels were inverse-normal transformed within each cohort to approximate a standard normal distribution. The null model incorporated covariates selected based on their significant associations with enzyme levels in preliminary linear regression models, including age, sex, body mass index (BMI), type 2 diabetes case/control status, and alcohol consumption (defined as any level of active alcohol intake). In the AADM cohort, we additionally adjusted for the first three genetic principal components (PCs) to account for fine-scale population structure, given the ancestral heterogeneity within this study. Variance components from the null GLMM were used in association testing to control for relatedness and population stratification. Per-cohort GWAS summary statistics were combined via fixed-effect inverse-variance weighted meta-analysis using METASOFT (v1.6). Genome-wide significance was defined as *P* < 5 × 10^−8^. A locus was considered novel if it was not previously reported in the GWAS Catalog for the corresponding liver enzyme phenotype and if the lead variant showed pairwise linkage disequilibrium (LD) *r*^2^ < 0.4 or was located more than 500 kb from known loci.

### Replication of novel loci

To evaluate the transferability of novel associations identified in our discovery analysis, we assessed replication in two independent datasets. First, we examined lead variants in the Uganda Genome Resource (UGR), a large African-ancestry cohort (Accession No: GCST009042^39^). Second, we queried summary statistics from a European-ancestry GWAS of liver enzymes conducted in the UK Biobank, accessed via the Pan-UK Biobank GWAS^50^. Replication was defined as a consistent direction of effect relative to the discovery analysis and a nominal association *P*-value < 0.05 in the replication dataset. This approach allowed us to explore both ancestry-specific and shared genetic effects across populations.

### Fine-mapping and functional annotation

To prioritize and interpret loci associated with liver enzymes, we performed fine-mapping and functional annotation using the Functional Mapping and Annotation (FUMA) platform (v1.5.2). Independent significant variants were first identified based on a genome-wide significance threshold (*P* < 5 × 10^−8^) and linkage disequilibrium (LD) criteria (pairwise *r*^2^ ≤ 0.6), using the African reference panel from the 1000 Genomes Project Phase 3. From these variants, lead variants were further refined using an LD threshold of *r*^2^ < 0.1, and risk loci were defined as clusters of lead variants within 250 kb and LD *r*^2^ ≥ 0.1. Variants were mapped to genes based on positional proximity (within 10 kb of annotated gene boundaries), liver-specific expression quantitative trait loci (eQTL) from GTEx v8, and chromatin interaction data. Enhancer annotations were incorporated from PsychENCODE, while liver-specific chromatin interaction mapping was conducted using Hi-C data (GSE87112) and enhancer-promoter correlations from the Roadmap Epigenomics liver dataset (E066), applying an FDR threshold of 1 × 10^−6^. We did not apply additional filtering based on Combined Annotation Dependent Depletion (CADD) scores, to avoid excluding non-coding but regulatory variants. To complement variant-level analyses, we performed gene-based association testing using MAGMA (v1.08), leveraging expression profiles from GTEx v8 (average log_2_TPM across tissues) to identify liver-enriched genes potentially associated with enzyme regulation but not captured by single variant associations.

To evaluate whether GWAS signals and gene expression regulation share a common causal variant, we conducted colocalization analyses using FastENLOC with liver-specific eQTL data from GTEx v8. FastENLOC estimates the regional posterior probability that the same underlying variant is responsible for both the GWAS association and eQTL signal within a specified tissue. We applied this approach to our summary statistics, focusing on the liver, to identify loci where regulatory variation may drive enzyme levels. Genes with high colocalization posterior probabilities were considered strong candidates for downstream functional validation.

To further resolve associated loci, we employed SuSiE (Sum of Single Effects) ^51^, a Bayesian variable selection method for fine-mapping based on summary-level GWAS data. SuSiE models the joint distribution of association signals while accounting for local linkage disequilibrium, producing credible sets of variants with associated posterior inclusion probabilities (PIPs). This framework enables the identification of likely causal variants within regions of complex LD structure and helps disentangle multiple association signals, thereby refining the interpretation of overlapping or pleiotropic genetic effects.

To elucidate the potential functional relevance of novel variants identified in our GWAS, we employed a multi-pronged approach that integrated regulatory annotation, phenome-wide association data, and gene enrichment analysis. First, we used HaploReg v4.1 to annotate each novel variant with regulatory features, leveraging the African-ancestry reference panel from the 1000 Genomes Project to capture population-specific linkage disequilibrium (LD) patterns. This analysis provided information on chromatin state segmentation (*e.g.*, promoter or enhancer annotations), histone modifications (*e.g.*, H3K4me1 and H3K27ac), DNase I hypersensitivity sites, altered transcription factor binding motifs, and LD proxies (*r*^2^ ≥ 0.6).

To evaluate the broader phenotypic relevance of the novel loci identified in our GWAS, we conducted phenome-wide association analyses (PheWAS) using summary statistics from the CIPHER Million Veteran Program PheWAS portal (https://phenomics.va.ornl.gov/pheweb/gia/afr/), which includes genome-wide data from individuals of genetically inferred African ancestry. For each novel lead variant, we extracted association estimates across a wide spectrum of phenotypes, ranging from metabolic and cardiovascular traits to liver-specific conditions and systemic inflammatory disorders. Analyses were conducted using an additive genetic model, and results were visualized as Manhattan plots to facilitate the identification of potential pleiotropic effects. Associations reaching a suggestive significance threshold (*P* < 1 × 10^−4^) were highlighted as indicative of cross-phenotype relevance. These PheWAS findings provided additional support for the clinical and biological relevance of novel variants, offering insight into their potential roles in complex disease mechanisms beyond liver enzyme regulation. To explore the biological pathways linked to these novel variants, we used the Database for Annotation, Visualization and Integrated Discovery (DAVID) for gene enrichment analysis. Genes identified through positional mapping, eQTL colocalization, or chromatin interaction were compiled and queried against Gene Ontology terms. We considered annotations statistically significant at a false discovery rate (FDR) < 0.05 under Biological Process and Molecular Function categories.

### Genetic correlation analysis

To quantify the extent of shared polygenic architecture among liver enzyme traits, we estimated pairwise genetic correlations using LD Score Regression (LDSC, v1.0.11). This method leverages GWAS summary statistics to evaluate the correlation between traits attributable to common genetic variants. We employed precomputed LD scores and regression weights based on the African-ancestry panel from the UK Biobank (UKBB.AFR) to ensure ancestry-matched linkage disequilibrium structure. Pairwise genetic correlations (*r*_g_) were computed using the ldsc.py --rg command, which performs a bivariate regression of the product of trait *z*-scores on LD scores across the genome. In addition, we obtained observed-scale SNP-based heritability (*h*^2^), LDSC intercepts, and genomic inflation factors (λ_GC_) for each liver enzyme trait. In instances where *r*_g_ estimates exceeded the theoretical bounds of ±1, these were retained and interpreted as indicative of strong polygenic sharing, recognizing that such values may arise due to sampling variability, especially when trait heritabilities are modest or sample sizes are limited.

## Supplementary Material

1 Supplementary Table 1. Genomic risk loci identified by FUMA for GWAS of ALP, ALT, AST, and GGT
Description: Genomic loci identified via FUMA from GWAS of four liver enzymes (ALP, ALT, AST, GGT). Each row corresponds to a unique genomic locus, with lead SNPs and associated statistics shown. Columns are: Phenotype, trait analyzed; GenomicLocus, numeric locus index assigned by FUMA; uniqID, unique variant identifier; rsID, dbSNP reference; chr, chromosome; pos, genomic position (base pair); p, *P*-value for the lead SNP; start/end, boundaries of the genomic locus; nSNPs, total number of SNPs in the locus after QC; nGWASSNPs, number of SNPs surpassing genome-wide significance in that locus; nIndSigSNPs, number of independently significant SNPs; IndSigSNPs, list of the independently significant variants; nLeadSNPs, total lead SNPs defining the locus at genome-wide significance; LeadSNPs, list of lead SNPs in that locus.

Supplementary Dataset 2. Lead SNPs for ALP, ALT, AST, and GGT identified by FUMA

Description: Independently significant SNPs for each locus, as determined by FUMA’s clumping approach, focusing on variants with lead signals for the four liver enzyme traits (ALP, ALT, AST, GGT). Columns are: Phenotype, trait under study; No, index for the entry; GenomicLocus, numeric locus index; uniqID, unique variant identifier; rsID, dbSNP reference; chr, chromosome; pos, genomic position (base pair); p, *P*-value for the lead SNP; nIndSigSNPs, number of independently significant SNPs; IndSigSNPs, list of those SNPs at each locus.

Supplementary Dataset 3. *TMEM64* associations from Open Targets Genetics

(https://genetics.opentargets.org/gene)

Description: *TMEM64* associations obtained from Open Targets Genetics, highlighting various biological and functional categories, as well as gene expression and dependency data. Columns are: association, biological/functional annotation; dataset, resource from which the association was derived (*e.g.*, Roadmap Epigenomics, COSMIC, Allen Brain Atlas, DepMap CRISPR); threshold value, significance or cutoff parameter; standardized value, a normalized metric of the observed association, if applicable.

Supplementary Dataset 4. *TP53TG1* associations from Open Targets Genetics

Description: *TP53TG1* associations obtained from Open Targets Genetics (https://genetics.opentargets.org/gene). Sources include epigenomic profiles, COSMIC cell-line data, Allen Brain Atlas, and DepMap CRISPR dependency. Columns are: association, biological or functional category; dataset, specific database or resource; threshold value, significance cutoff or threshold; standardized value, normalized metric of the observed association (if applicable).

This is a list of supplementary files associated with this preprint. Click to download.

• SupplementaryDatasets.xlsx

## Figures and Tables

**Table 1.** Significant loci in GWAS of liver enzymes in individuals of African ancestry

rsID	Gene	Chromosome :position	Effect/ Ref. allele	Effect allele Freq.	Consequence	Effect (Beta)	SE	*P*	Studies Replicated*
Alkaline phosphatase (ALP)									
rs6666477	*ALPL*	1:21870681	C/T	0.380	intronic	0.0652	0.0080	3.00 × 10^−16^	^13,18,19^
**rs144252352**	***DIRC3***	**2:218399896**	**A/G**	**0.007**	**intergenic**	**−0.2487**	**0.0432**	**8.69 × 10^−09^**	**Novel**
**rs140363270**	***ST3GAL6-AS1***	**3:98431154**	**C/T**	**0.055**	**intergenic**	**0.1027**	**0.0170**	**1.63 × 10^−09^**	**Novel**
rs537496263	*MRS2*	6:24398376	A/AT	0.098	intergenic	−0.1194	0.0144	1.00 × 10^−16^	^3,13,18-20^
rs58737329	*CD36*	7:80188082	A/G	0.079	intronic	−0.1455	0.0176	1.00 × 10^−16^	^21^
rs10099512	*RP11-115J16.1*	8:9178821	C/G	0.227	ncRNA intronic	−0.0773	0.0094	1.00 × 10^−16^	^4,13,18,19,22^
rs904018	*GATA4*	8:11616516	C/T	0.458	UTR3	−0.0479	0.0078	8.78 × 10^−10^	*GATA4* ^18^
**rs78306989**	***TMEM64***	**8:91695044**	**A/G**	**0.069**	**intronic**	**0.1071**	**0.0158**	**1.30 × 10^−11^**	**Novel**
rs582118	*ABO*	9:136145471	A/G	0.477	ncRNA intronic	0.0719	0.0088	2.00 × 10^−16^	*ABO* ^3,20,23^
rs7910927	*JMJD1C*	10:65138910	G/T	0.284	intronic	0.0618	0.0083	1.21 × 10^−13^	^19^
rs3817640	*IFITM5*	11:297970	C/T	0.168	downstream	0.0759	0.0105	5.99 × 10^−13^	*IFITM5* ^3,22,24^
**rs9550655**	***CRYL1***	**13:21063974**	**C/T**	**0.036**	**intronic**	**−0.1068**	**0.0174**	**8.59 × 10^−10^**	**Novel**
rs11647069	*PMFBP1*	16:72155237	C/T	0.214	intronic	−0.0587	0.0093	3.28 × 10^−10^	*PMFBP1* ^3,4,19^
rs116089734	*ASGR1*	17:7075351	A/C	0.031	intergenic	−0.1442	0.0254	1.31 × 10^−08^	*ASGR1* ^19,22,24^
rs34182948	*NRTN*	19:5824438	G/GT	0.256	intronic	0.0809	0.0098	1.00 × 10^−16^	^3^
rs34954997	*APOC1*	19:45417638	C/CTTCG	0.319	UTR5	0.0722	0.0091	2.40 × 10^−15^	^18,19,24^
rs281377	*FUT2*	19:49206603	C/T	0.268	exonic	0.0726	0.0089	3.00 × 10^−16^	^13^
Alanine transaminase (ALT)									
rs137964419	*KIAA1324L*	7:86893089	A/G	0.017	intergenic	0.2396	0.0406	1.00 × 10^−16^	^25,26^
**rs138797771**	***IMMP2L***	**7:110842764**	**C/T**	**0.006**	**intronic**	**0.2446**	**0.0435**	**1.88 × 10^−8^**	**Novel**
rs2721150	*PPP1R16A*	8:145724973	C/G	0.455	ncRNA exonic	−0.0653	0.0079	1.00 × 10^−16^	^18,25,27^
rs4753126	*PANX1*	11:93862020	C/T	0.437	upstream	0.0440	0.0079	2.34 × 10^−8^	*PANX1* ^20^
rs738408	*PNPLA3*	22:44324730	C/T	0.118	exonic	0.2396	0.0406	1.29 × 10^−13^	^23,26,28-32^
Aspartate transaminase (AST)									
rs576738951	*CROT*	7:87012854	C/CAGG	0.017	intronic	0.1918	0.0339	1.56 × 10^−8^	^25,26^
**rs7086539**	***C1QL3***	**10:16583146**	**G/T**	**0.280**	**intergenic**	**0.0512**	**0.0087**	**3.62 × 10^−9^**	**Novel**
**rs56331215**	***MRC1L1***	**10:17875816**	**A/T**	**0.008**	**exonic**	**0.0626**	**0.0089**	**2.44 × 10^−12^**	**Novel**
rs71497225	*MRC1*	10:18138665	C/G	0.413	exonic	−0.0794	0.0118	1.77 × 10^−11^	*MRC1* ^22,33^
rs12485100	*PNPLA3*	22:44325516	G/T	0.110	intronic	0.1918	0.0339	6.60 × 10^−13^	^26,27,33,34^
Gamma-glutamyl transferase (GGT)									
rs6675677	*USP48*	1:22026301	A/G	0.489	intronic	−0.0687	0.0122	1.96 × 10^−8^	*USP48* ^18^
**rs864064**	***PDE4D***	**5:59400110**	**G/T**	**0.321**	**intronic**	**0.0752**	**0.0133**	**1.47 × 10^−8^**	**Novel** ^18,20,21,24,28,30,35-39^
rs199882393	*GGT5*	22:24608818	T/TAAAA	0.048	intergenic	0.2632	0.0320	1.00 × 10^−16^	
**rs2236005**	***MYO18B***	**22:26422980**	**A/G**	**0.089**	**exonic**	**0.1100**	**0.0174**	**2.32 × 10^−10^**	**Novel**

* All SNPs that were genome-wide significant and those in high LD with the lead variant (*r*^2^>0.8) were searched in the NHGRI-EBI GWAS Catalog. The gene was searched if the variant or those in LD with it were missing. Associations that have not been reported are shown in bold. Genomic coordinates are based on the GRCh37/hg19 reference genome build.

## Data Availability

The datasets from the Africa America Diabetes Mellitus (AADM) study and the Howard University Family Study (HUFS) are available from C.N.R. upon reasonable request and subject to institutional review board (IRB) approval and participant consent agreements. Data from the CARDIA (accession no. phs000285) and MESA (accession no. phs000209) can be accessed through dbGaP (Database of Genotypes and Phenotypes) upon submission and approval of an authorized data access request. Summary-level genome-wide association statistics generated from this study have been deposited in the GWAS Catalog. Analysis scripts and computational workflows are publicly available at: https://github.com/rmogire.
