# Supplementary material for: Multi-cohort genome-wide association analyses reveal loci underlying circulating liver enzyme levels in African-ancestry populations
Source: Res Sq. 2025 Jul 24:rs.3.rs-6941679. Preprint. [Version 1] doi: 10.21203/rs.3.rs-6941679/v1 (PMC12747275; doi:10.21203/rs.3.rs-6941679/v1)
Supplement: 1 — Supplementary Table 1. Genomic risk loci identified by FUMA for GWAS of ALP, ALT, AST, and GGT Description: Genomic loci identified via FUMA from GWAS of four liver enzymes (ALP, ALT, AST, GGT). Each row corresponds to a unique genomic locus, with lead SNPs and associated statistics shown. Columns are: Phenotype, trait analyzed; GenomicLocus, numeric locus index assigned by FUMA; uniqID, unique variant identifier; rsID, dbSNP reference; chr, chromosome; pos, genomic position (base pair); p, P-value for the lead SNP; start/end, boundaries of the genomic locus; nSNPs, total number of SNPs in the locus after QC; nGWASSNPs, number of SNPs surpassing genome-wide significance in that locus; nIndSigSNPs, number of independently significant SNPs; IndSigSNPs, list of the independently significant variants; nLeadSNPs, total lead SNPs defining the locus at genome-wide significance; LeadSNPs, list of lead SNPs in that locus. [file NIHPPrs6941679v1-supplement-1.pdf]

Supplementary Table 1. Characteristics of study cohorts

	**Discovery cohorts**						**Replication cohort**
	**AADM**	**HUFS**	**CARDIA**	**MESA**	**UK Biobank (African ancestry)**	**African Americans (All of Us)**	**Uganda Genome Resource**
Median age (IQR, years)	52.0 (42.0, 61.0)	42.0 (24.0, 51.0)	24.0 (21.0, 28.0)	62.0 (53.0, 70.0)	50.0 (45.0, 58.0) y	61.0 (50.0, 69.0) y	30 (17, 46) y
Sex: females (%)	3380/5537 (61.04%)	1,279/2100 (60.90%)	988/1146 (58.77%)	906/1677 (54.03%)	3,393/8,151 (41.6%)	6,458/8,935 (72.28%)	4,404/7,833 (56.22%)
Type 2 diabetes (%)	2704 (48.84%)	234 (11.14%)	15 (0.90%)	286 (17.12%)	n/a	n/a	102/7,833 (1.3%)
BMI (IQR)	26.2 (22.7, 30.2)	28.2 (23.8, 34.3)	24.1 (21.5, 28.1)	29.4 (26.1, 33.5)	28.7 (25.7, 32.3)	32.1 (27.1, 38.4)	n/a
Alcohol intake (yes)*	265 (4.79%)	291 (13.86%)	1352 (80.62%)	543 (32.79%)	n/a	n/a	2793/7,833 (35.66%)
Alkaline phosphatase (u/l)	64.0 (51.0, 80.0) (n=1123)	73.0 (59.0, 92.0) (n=1848)	52.0 (43.0, 65.0) (n=1146)	n/a	n/a	(n=22507)	71.3 (92.5, 144.1)
Aspartate aminotransferase (u/l)	13.0 (9.0, 19.0) (n=790)	n/a	23.0 (18.0, 29.0) (n=1146)	n/a	(n=6180)	(n=22276)	14.0 (17.8, 22.9)
Alanine aminotransferase (u/l)	8.0 (5.0, 13.0) (n=1200)	10.0 (6.0, 15.0) (n=1935)	n/a	n/a	(n=6180)	(n=19674)	21.2 (25.1, 30.4)
Gamma-glutamyl transferase (u/l)	n/a	n/a	9.0 (6.0, 14.0) (n=1146)	9.5 (6.1, 15.3) (n=1382)	(n=6212)	(n=2248)	13.5 (18.7–28.0)
Study Site	Ghana, Nigeria, Kenya	USA	USA	USA	United Kingdom	USA	Uganda
Study design	Multicenter, focused on genetic risk factors for T2D	Population-based family study	Multicenter, longitudinal cohort of young adults	Multi-ethnic prospective cohort	Population-based cohort	Nation-wide program recruiting diverse participants	Population-based cohort
Genotyping platform	Affymetrix Axiom® PanAFR / Illumina MEGA	Affymetrix 6.0 / Illumina MEGA	Affymetrix 6.0	Affymetrix 6.0	UK Biobank Axiom™ Array	Illumina NovaSeq 6000 whole genome sequencing	Illumina 2.5M
Number of variants in chip	~900,000 (chip), imputed to >10M	~900,000 (chip), imputed to >10M	~900,000 (chip), imputed to >10M	~900,000 (chip), imputed to >10M	850,000 (chip-based), imputed to ~96M	Whole genome sequence (30×)	~2.5 million

Median estimates are based on all participants with data. Abbreviations; IQR, interquartile range; n/a, not available. [§]The number of participants in each study included in the GWAS was based on the availability of genotype and phenotype data.

Supplementary Table 2. Genome-wide significant variants and loci reported in past studies

rsID	Gene	Chromosome :position	Effect/ Ref. allele	Effect allele Freq.	Variants that have been reported *
**Alkaline phosphatase (ALP)**					
rs6666477	*ALPL*	1:21870681	C/T	0.380	rs1976403[13], rs3200254[18], rs1780329[18], rs1697405[19]
**rs144252352**	***DIRC3***	**2:218399896**	**A/G**	**0.007**	**Novel**
**rs140363270**	***ST3GAL6-AS1***	**3:98431154**	**C/T**	**0.055**	**Novel**
rs537496263	*MRS2*	6:24398376	A/AT	0.098	rs9467148[19], rs9467191[20], rs7765515, rs16889440, rs73727344 and rs2235501[18], rs7765441[3], rs1883415 [13]
rs58737329	*CD36*	7:80188082	A/G	0.079	rs3211938[21]
rs10099512	*RP11-115J16.1*	8:9178821	C/G	0.227	rs983309 [18], rs6984305 [19], rs4841132[22], rs4240624 and rs1461729[4], rs6984305*[13]
rs904018	*GATA4*	8:11616516	C/T	0.458	*GATA4*[18]
**rs78306989**	***TMEM64***	**8:91695044**	**A/G**	**0.069**	**Novel**
rs582118	*ABO*	9:136145471	A/G	0.477	*ABO*[3,20,23]
rs7910927	*JMJD1C*	10:65138910	G/T	0.284	rs1935*3
rs3817640	*IFITM5*	11:297970	C/T	0.168	*IFITM5*[3,22,24]
**rs9550655**	***CRYL1***	**13:21063974**	**C/T**	**0.036**	**Novel**
rs11647069	*PMFBP1*	16:72155237	C/T	0.214	*PMFBP1*[3,4,19]
rs116089734	*ASGR1*	17:7075351	A/C	0.031	*ASGR1*[19,22,24]
rs34182948	*NRTN*	19:5824438	G/GT	0.256	rs10409772[3]
rs34954997	*APOC1*	19:45417638	C/CTTCG	0.319	rs4420638 and rs57940795[18], rs5112 [19], rs492602[24]
rs281377	*FUT2*	19:49206603	C/T	0.268	rs281377[13] (actual SNP)
**Alanine transaminase (ALT)**					
rs137964419	*KIAA1324L*	7:86893089	A/G	0.017	rs115038698[25], rs61730509[26]
**rs138797771**	***IMMP2L***	**7:110842764**	**C/T**	**0.006**	**Novel**
rs2721150	*PPP1R16A*	8:145724973	C/G	0.455	rs115230836[27], rs201082887[25], rs35842750 and rs4244612[18]
rs4753126	*PANX1*	11:93862020	C/T	0.437	*PANX1*[20]
rs738408	*PNPLA3*	22:44324730	C/T	0.118	rs738408-actual SNP[28],rs738409[29], rs3747207[30], rs12484801[31], rs12483959[32], rs4823173[26], rs2281135[23]
**Aspartate transaminase (AST)**					
rs576738951	*CROT*	7:87012854	C/CAGG	0.017	rs147423000* and rs144656711[26], rs115038698[25]*
**rs7086539**	***C1QL3***	**10:16583146**	**G/T**	**0.280**	**Novel**
**rs56331215**	***MRC1L1***	**10:17875816**	**A/T**	**0.008**	**Novel**
rs71497225	*MRC1*	10:18138665	C/G	0.413	*MRC1*[22,33]
rs12485100	*PNPLA3*	22:44325516	G/T	0.110	rs738409 and rs738408[26,33], rs3747207[27], rs4823173[34]
**Gamma-glutamyl transferase (GGT)**					
rs6675677	*USP48*	1:22026301	A/G	0.489	*USP48*[18]
**rs864064**	***PDE4D***	**5:59400110**	**G/T**	**0.321**	**Novel**
rs199882393	*GGT5*	22:24608818	T/TAAAA	0.048	rs73396312[35], rs61737674[18], rs9306395[20], rs737128 and rs5751901[36], rs4820599[18], rs6004193 and rs5751902[37], rs743371and rs2073398[24], rs2006092[30], rs3859862[38], rs2330805[28], rs1807629[38], rs57719575[21], rs115231893[39]
**rs2236005**	***MYO18B***	**22:26422980**	**A/G**	**0.089**	**Novel**

Genomic coordinates are based on the GRCh37/hg19 reference genome build.

Supplementary Table 3. Replication of novel loci in GWAS of liver enzymes in Ugandans and Europeans (UK Biobank)

					Current GWAS			Replication GWAS				
**RSID**	**Nearest Gene**	**Chromosome : position**	**Effect/ Ref. allele**	**Effect allele Freq.**	**Effect (Beta)**	**SE**	***P***	**Ancestry**	**Allele Freq.**	**Effect (Beta)**	**SE**	***P***
**Alkaline phosphatase (ALP)**												
rs144252352	*DIRC3*	2:218399896	A/G	0.0068	-0.249	0.0432	8.69×10^{-09}	AFR-UG	0.0048	n/a	n/a	n/a
								EUR-UKB	n/a	n/a	n/a	n/a
rs140363270	*ST3GAL6 -AS1*	3:98431154	C/T	0.055	0.103	0.0170	1.63×10^{-09}	AFR-UG	0.044	0.118	0.0447	0.0084
								EUR-UKB	0.00013	0.0546	0.132	0.68
rs78306989	*TMEM64*	8:91695044	A/G	0.069	0.107	0.0158	1.30×10^{-11}	AFR-UG	0.090	0.0217	0.0318	0.50
								EUR-UKB	0.00044	-0.0990	0.071	0.16
rs9550655	*CRYL1*	13:21063974	C/T	0.036	-0.107	0.0174	8.59×10^{-10}	AFR-UG	0.027	0.0562	0.0562	0.32
								EUR-UKB	0.23	-0.00243	0.003	0.43
**Alanine transaminase (ALT)**												
rs138797771	*IMMP2L*	7:110842764	C/T	0.0061	0.245	0.0435	1.88×10^{-08}	AFR-UG	0.0020	n/a	n/a	n/a
								EUR-UKB	n/a	n/a	n/a	n/a
**Aspartate transaminase (AST)**												
rs7086539	*C1QL3*	10:16583146	G/T	0.28	0.0512	0.00867	3.62×10^{-09}	AFR-UG	0.33	0.0571	0.020	0.0040
								EUR-UKB	0.28	-0.00189	0.00273	0.49
rs56331215	*MRC1L1*	10:17875816	A/T	0.008	0.0626	0.0089	2.44×10^{-12}	AFR-UG	n/a	n/a	n/a	n/a
								EUR-UKB	n/a	n/a	n/a	n/a
**Gamma-glutamyl transferase (GGT)**												
rs864064	*PDE4D*	5:59400110	G/T	0.32	0.0752	0.0133	1.47×10^{-8}	AFR-UG	0.27	-0.0149	0.0204	0.46
								EUR-UKB	0.47	0.000498	0.00252	0.84
rs2236005	*MYO18B*	22:26422980	A/G	0.089	0.1100	0.0174	2.32×10^{-10}	AFR-UG	0.11	-0.0149	0.0289	0.61
								EUR-UKB	0.15	0.0017	0.00343	0.62

n/a data for the SNP was not available;dash (-) implies the GWAS was not reported. EUR is Europeans in UK Biobank; AFR are Africans in the Uganda Genome Resource. Abbreviations; MVP, the Million Veteran Program: UG, Uganda Genome Resource; UKB, UK Biobank. Estimates for the mean inverse transformed levels were reported for the MVP data. Genomic coordinates are based on the GRCh37/hg19 reference genome build.

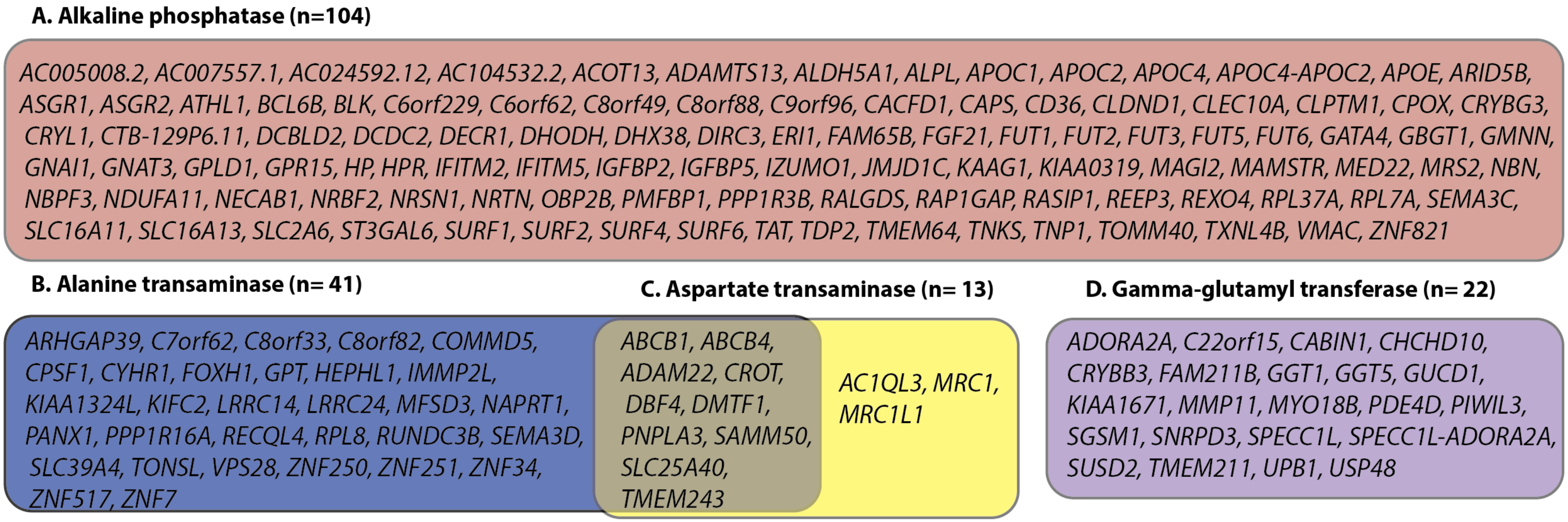

Supplementary Figure 1. Summary of overlap among genes mapped to loci associated with alkaline phosphatase, alanine transaminase, aspartate transaminase and gamma-glutamyl transferase. Overlap was determined using FUMA (https://fuma.ctglab.nl/) based on positional, eQTL, and chromatin interaction mapping approaches.

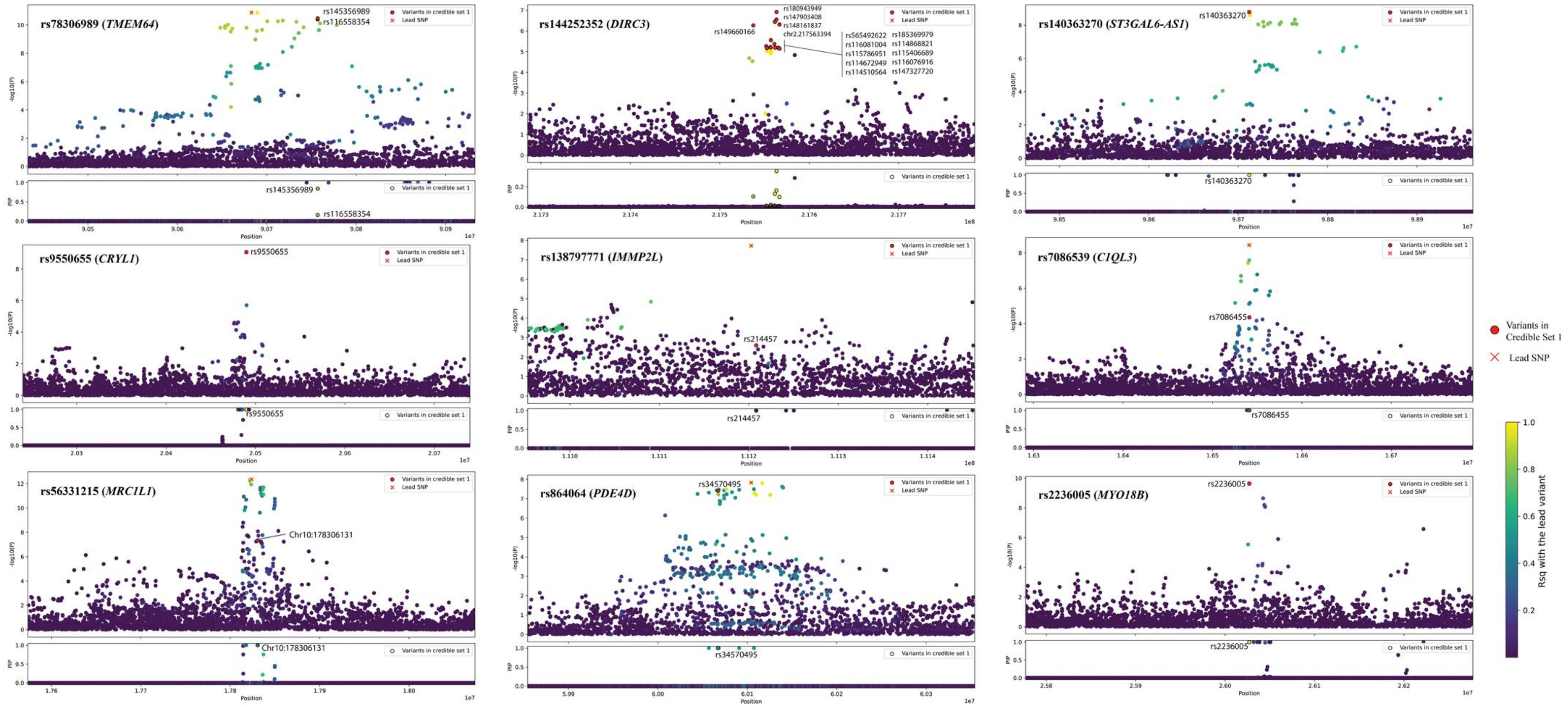

Supplementary Figure 2. SuSiE fine-mapping plots for nine novel loci associated with liver enzymes in individuals of sub-Saharan African ancestry. Each panel displays $-\log_{10}(P)$ values (top) and posterior inclusion probabilities (PIP; bottom) as a function of genomic position. Variants are colored by LD (r^2) with the lead SNP. SNPs within the 95% credible set are highlighted and annotated by genomic position. Genomic coordinates are based on the GRCh38 reference genome build.

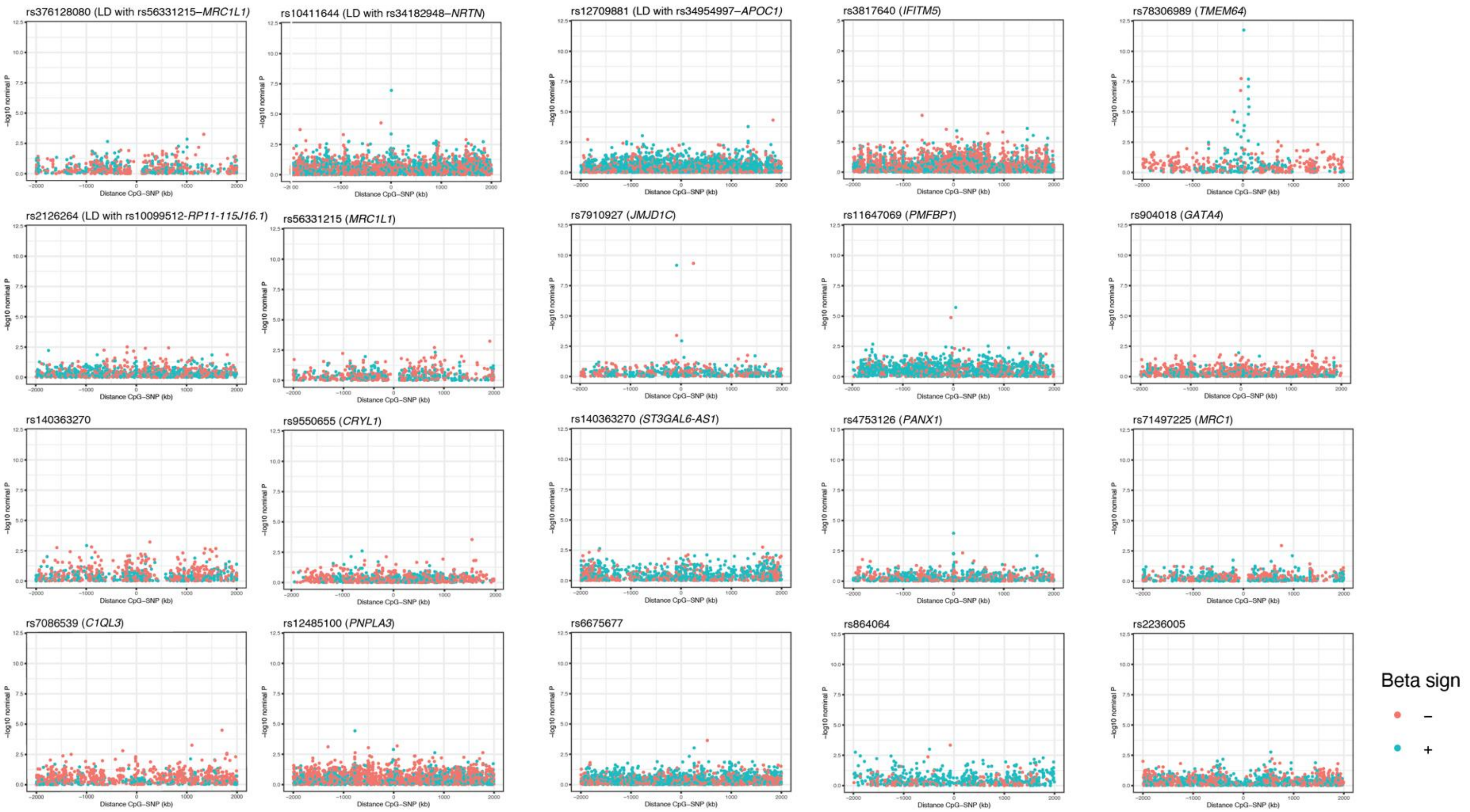

Supplementary Figure 3. ***Cis*-mQTL landscapes of eight GWAS liver enzyme loci in primary African-ancestry hepatocytes.** Each panel displays the association of a lead GWAS variant (or its $r^2 \geq 0.80$ proxy, indicated in the title) with DNA-methylation levels at all CpG probes located within ± 2 Mb of the SNP. The *x*-axis gives the physical distance between CpG and SNP (kb), while the *y*-axis shows the strength of association as $-\log_{10}$(nominal *P*). Points are color-coded by the direction of the regression coefficient (β) returned by QTLtools: turquoise for positive effects (allele increases methylation) and red for negative effects (allele decreases methylation).

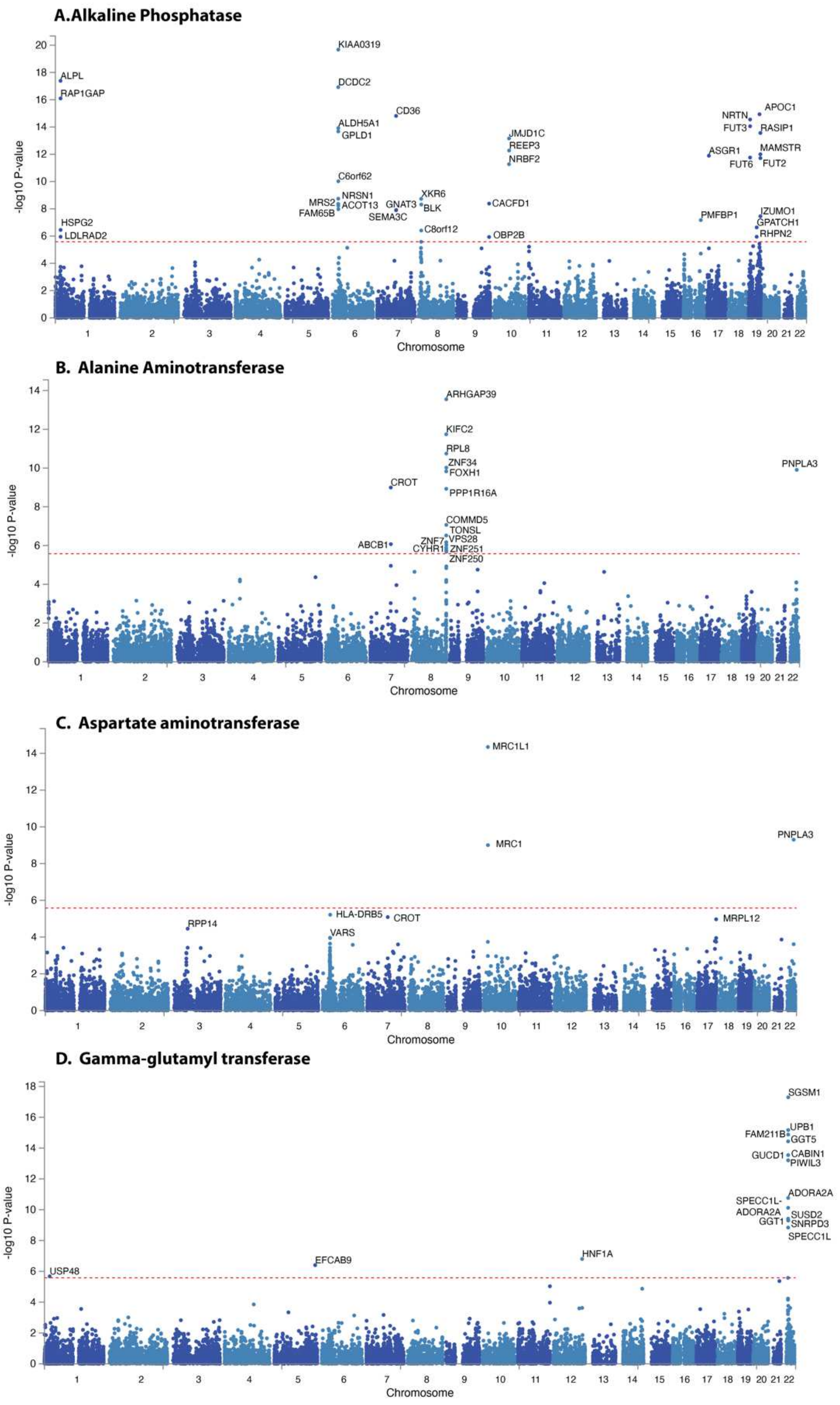

Supplementary Figure 4: Gene-based association test results. Manhattan plots for A) alkaline phosphatase, B) alanine transaminase, C) aspartate transaminase, and D) gamma-glutamyl transferase.

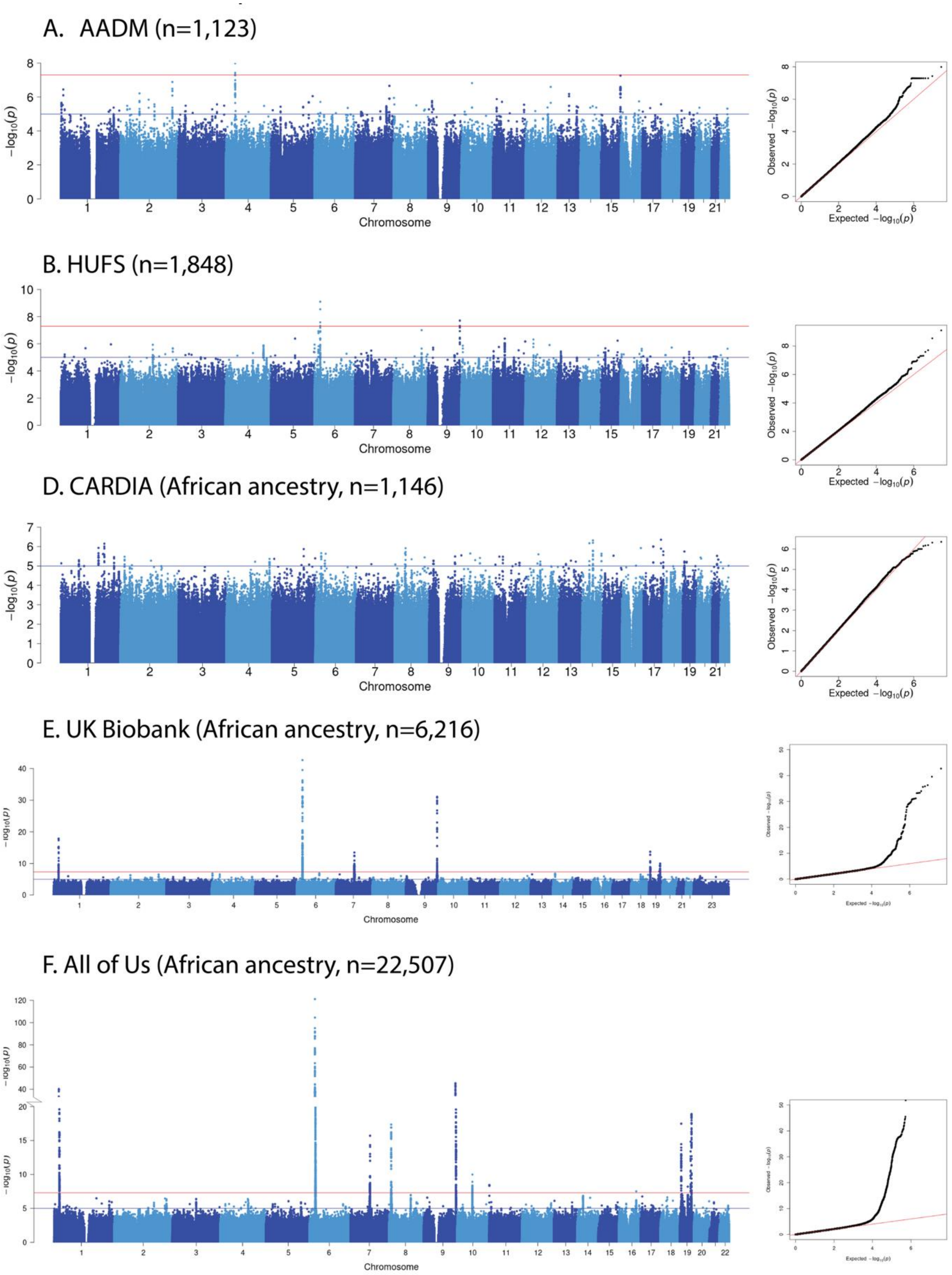

Supplementary Figure 5. Manhattan and quantile-quantile plots of GWAS of alkaline phosphatase levels by cohort.

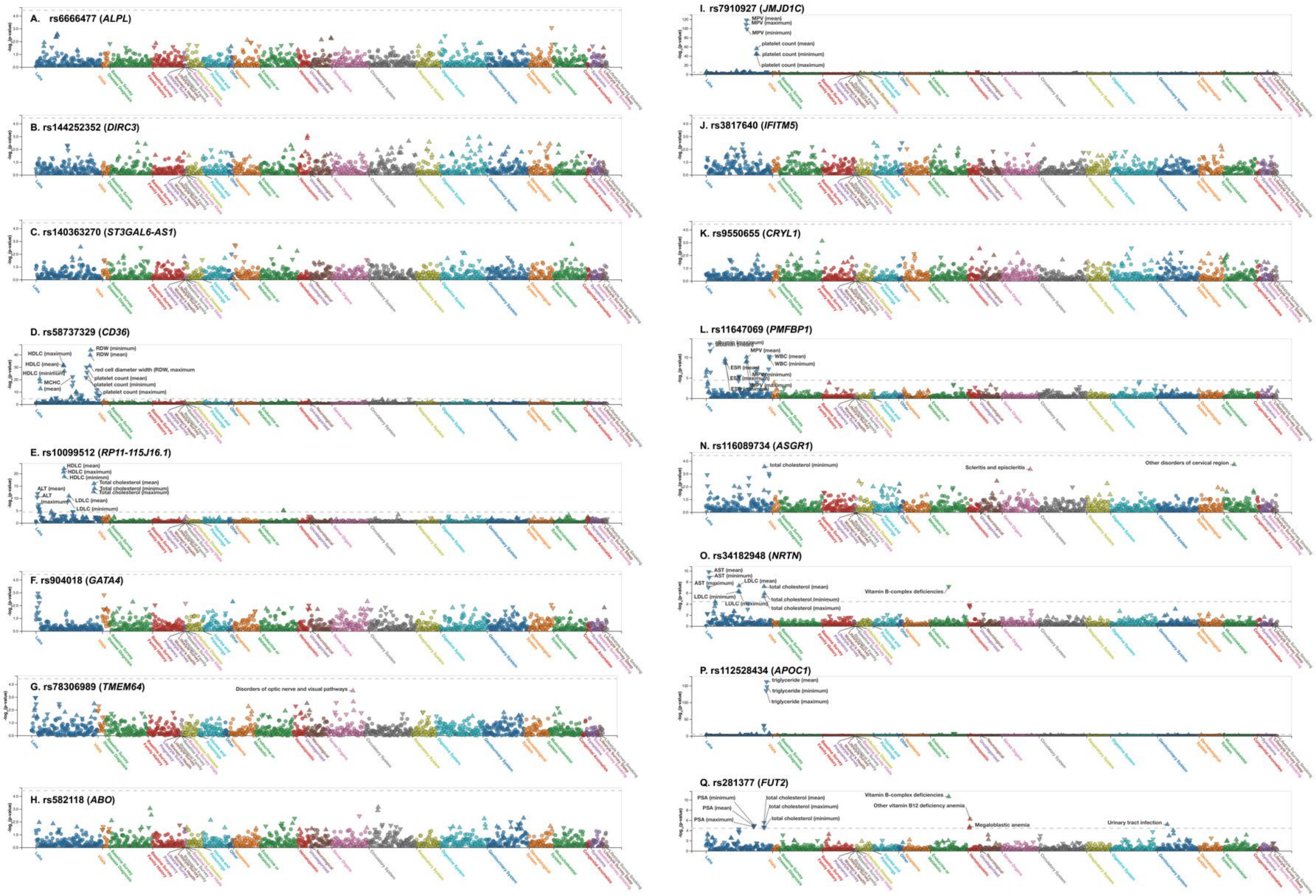

Supplementary Figure 6. Genome-wide PheWAS of novel loci variants associated with alkaline phosphatase. Analyses were conducted in the Million Veteran Program. Quantitative traits were inverse-normal transformed. Abbreviations: MCHC, mean corpuscular hemoglobin concentration; RDW red cell diameter width; HDLC, high density lipoprotein cholesterol (maximum). Rs112528434 is presented as a proxy for rs34954997. Rs537496263 and rs34954997 are missing.

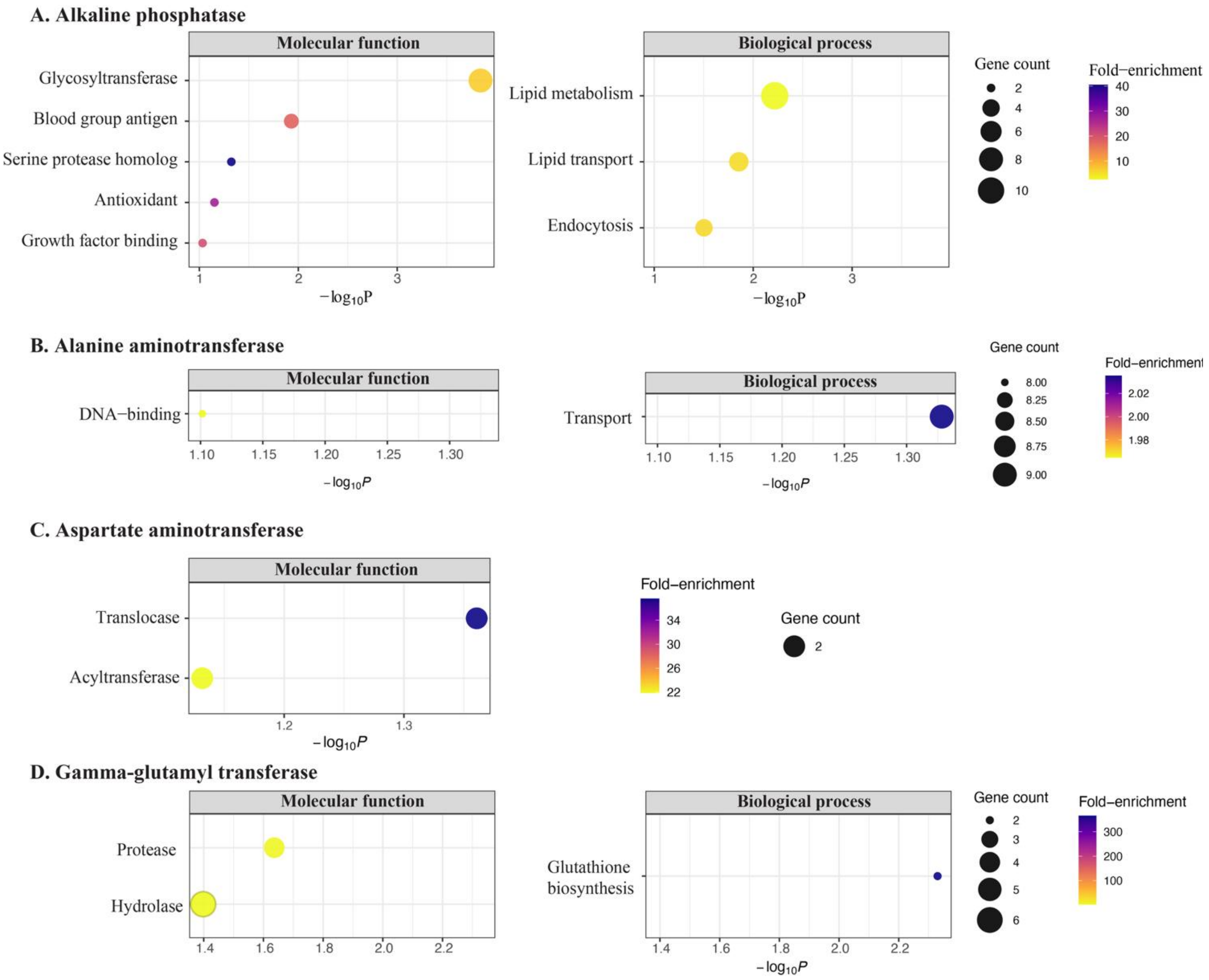

Supplementary Figure 7. DAVID-based Gene Ontology enrichment analysis for genes associated with ALP (A), ALT (B), AST (C), or GGT (D). For each enzyme panel the results are split into DAVID's Biological process (left) and Molecular function (right) - Keyword categories. Bubble area scales with the number of input genes annotated to the term, and bubble colour maps to fold-enrichment

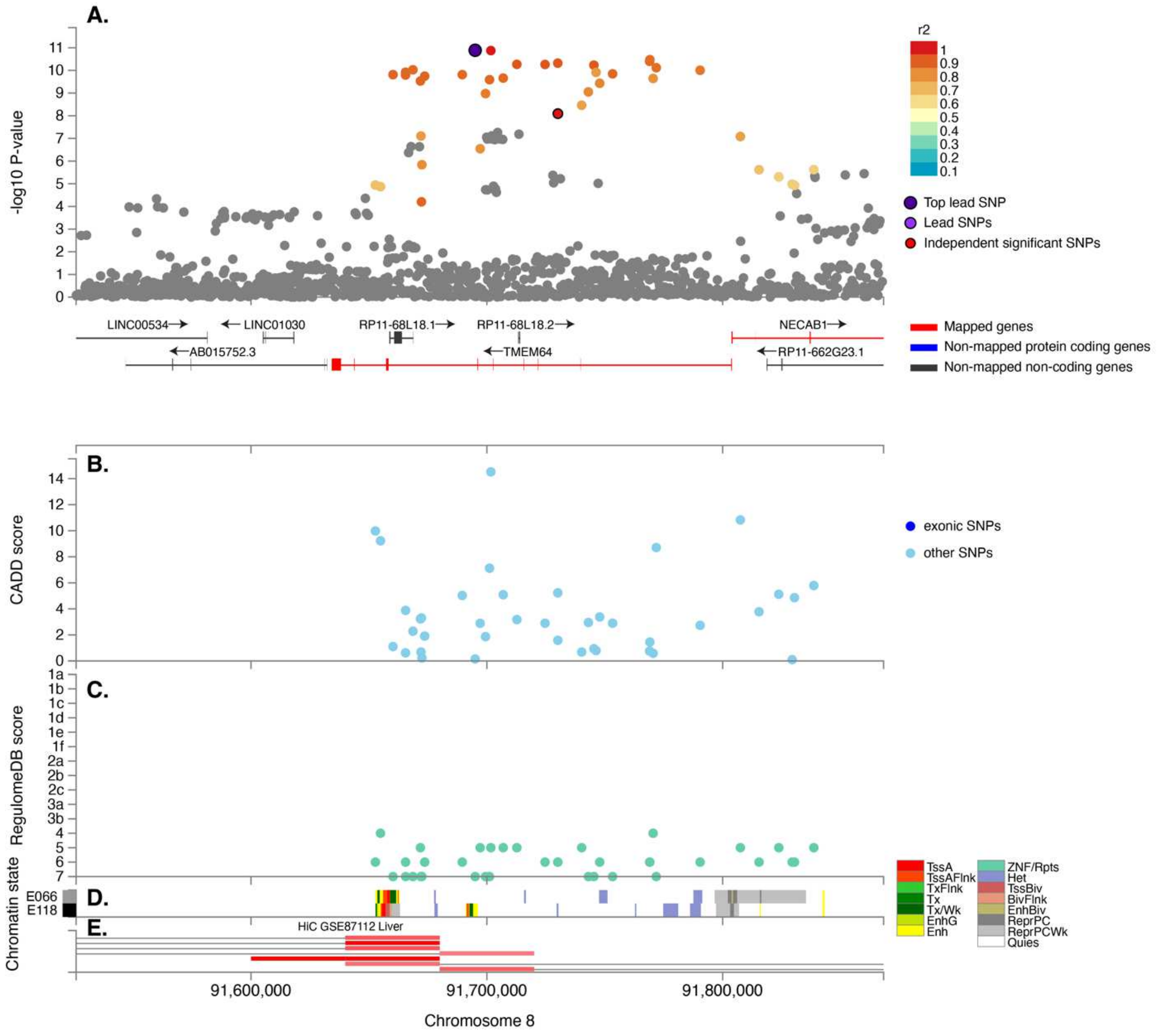

Supplementary Figure 8: Integrative functional annotation of the *TMEM64* locus. A) This panel shows regional GWAS association signals on chromosome 8, with SNPs colored by linkage disequilibrium (r^2) relative to the lead SNP. B) This panel displays Combined Annotation Dependent Depletion (CADD) scores. A subset of SNPs shows moderately high CADD scores, indicating potential deleteriousness. C) This panel shows functional impact scores based on RegulomeDB annotations. D) This panels illustrates chromatin state annotations from the Roadmap Epigenomics Project, highlighting active promoter and enhancer marks overlapping the associated SNPs in liver. While no significant eQTLs were detected in the selected tissues, the convergence of statistical association and epigenomic signals suggests a putative regulatory role for TMEM64 in liver-related function.

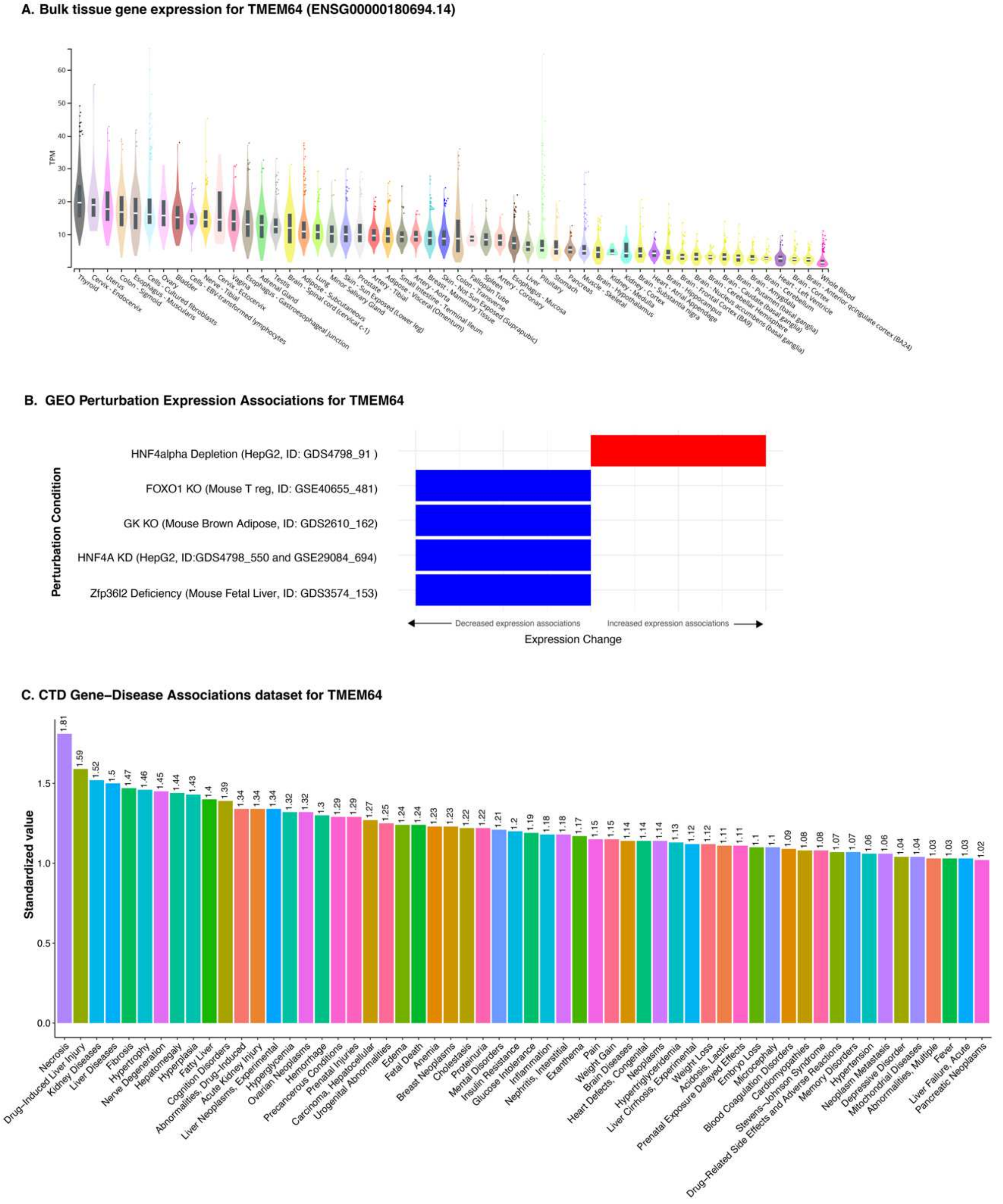

Supplementary Figure 9. A) Bulk tissue gene expression of *TMEM64* from the GTEx dataset. B) GEO perturbation expression associations for *TMEM64*. C) CTD Gene–Disease Associations dataset showing the top diseases associated with *TMEM64*. Gene–disease and gene expression association data were obtained from Harmonizome 3.0 (https://maayanlab.cloud/Harmonizome/); the complete dataset is provided in Supplementary Dataset 4. Bulk tissue gene expression data were retrieved from the GTEx Portal (https://gtexportal.org/). Bar labels in the GEO Perturbation Expression Associations plot include GEO accession IDs.

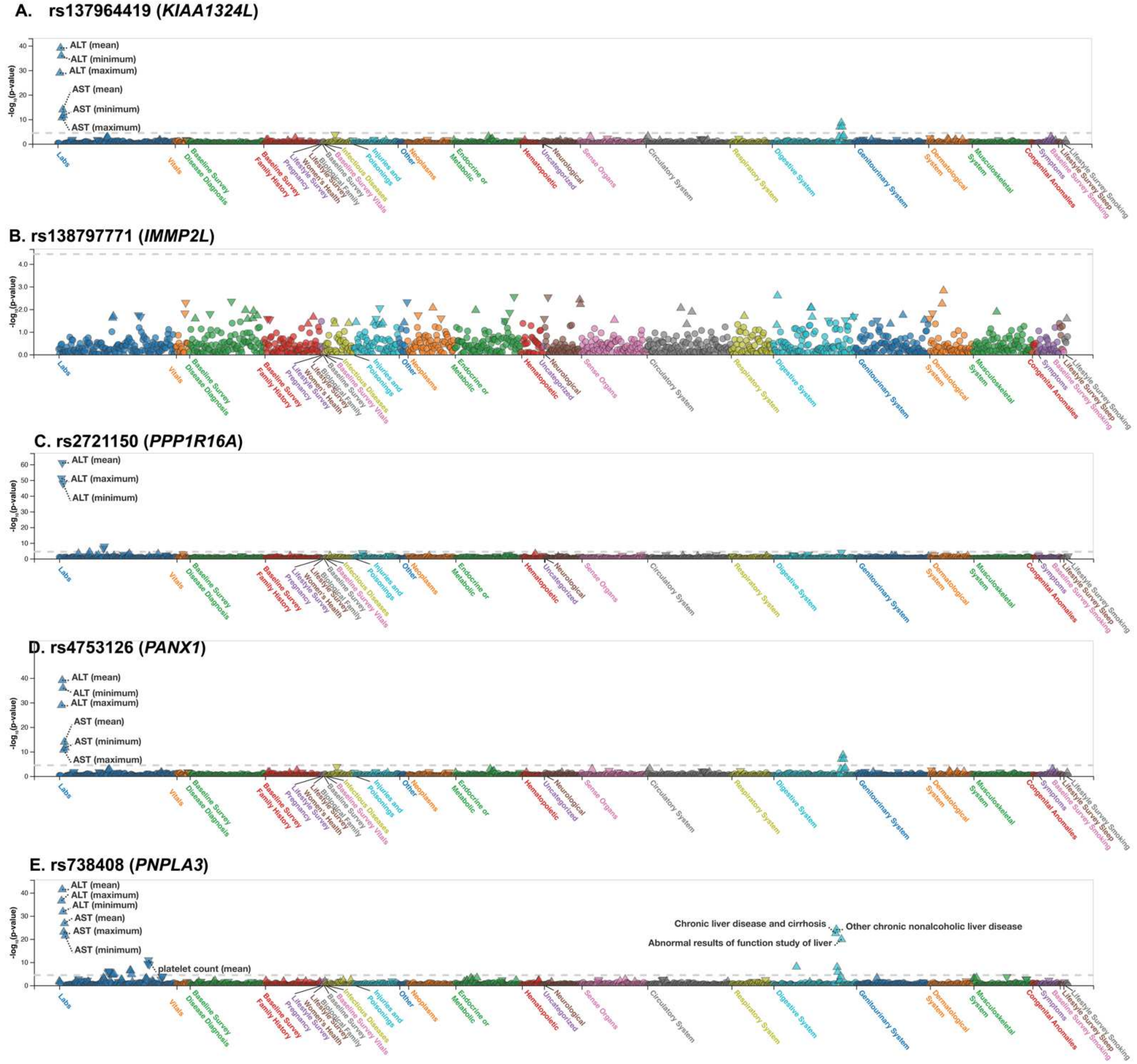

Supplementary Figure 10. PheWAS findings of ALT loci variants. Analyses were conducted in the Million Veteran Program. Quantitative traits were inverse-normal transformed.

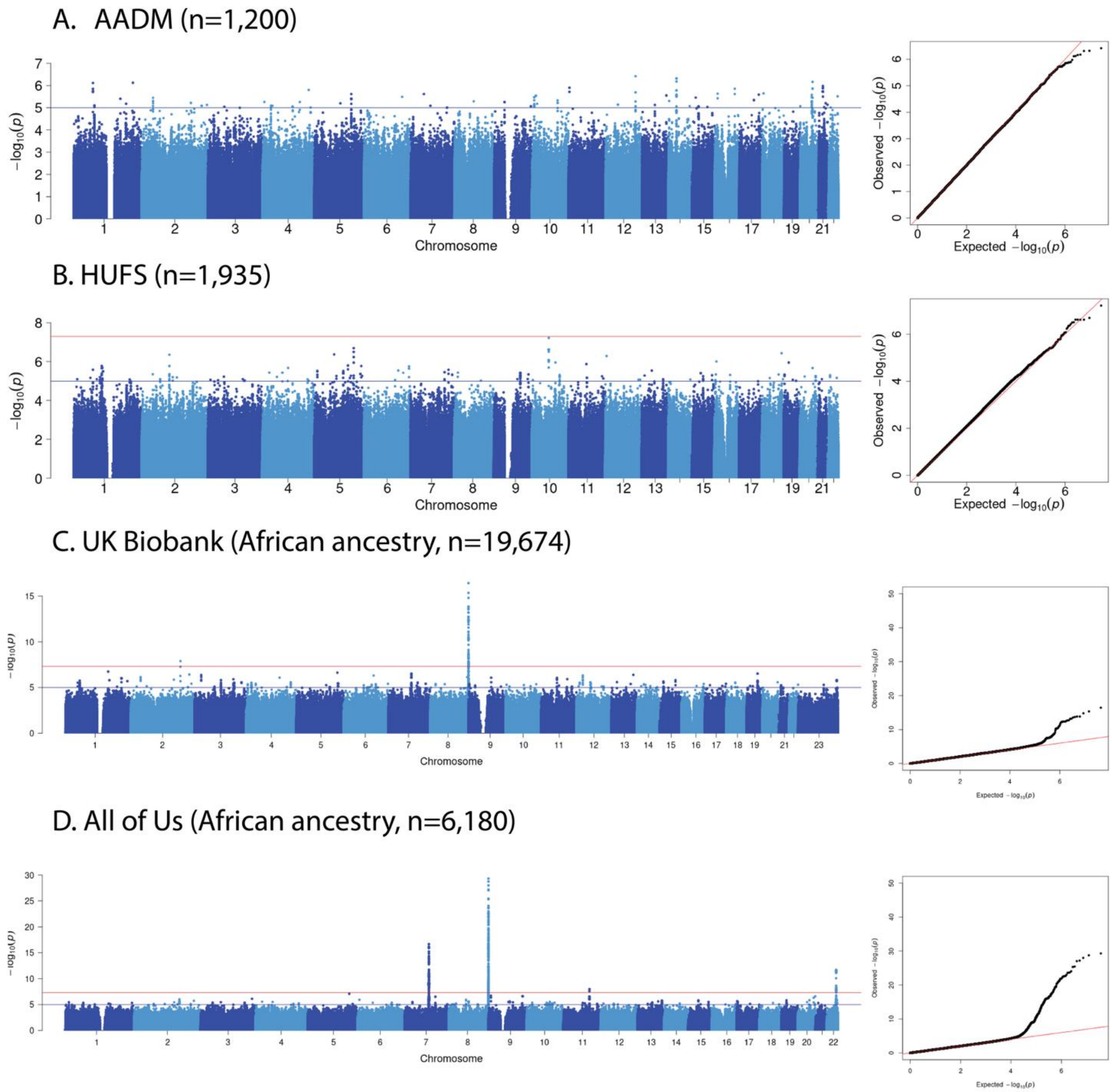

Supplementary Figure 11. Manhattan and quantile-quantile plots of GWAS of alanine aminotransferase levels by cohort.

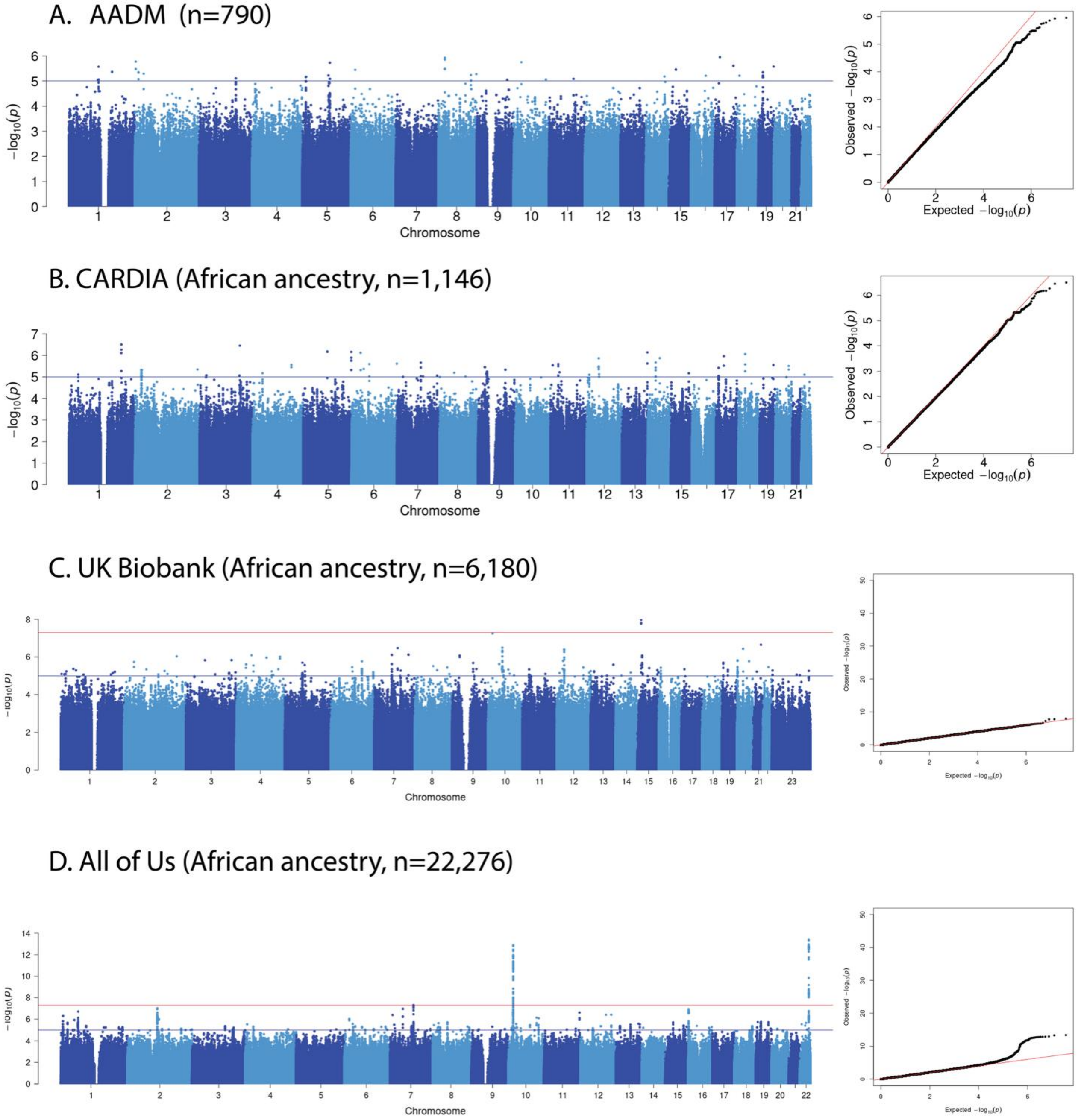

Supplementary Figure 12. Manhattan and quantile-quantile plots of GWAS of aspartate transaminase levels by cohort.

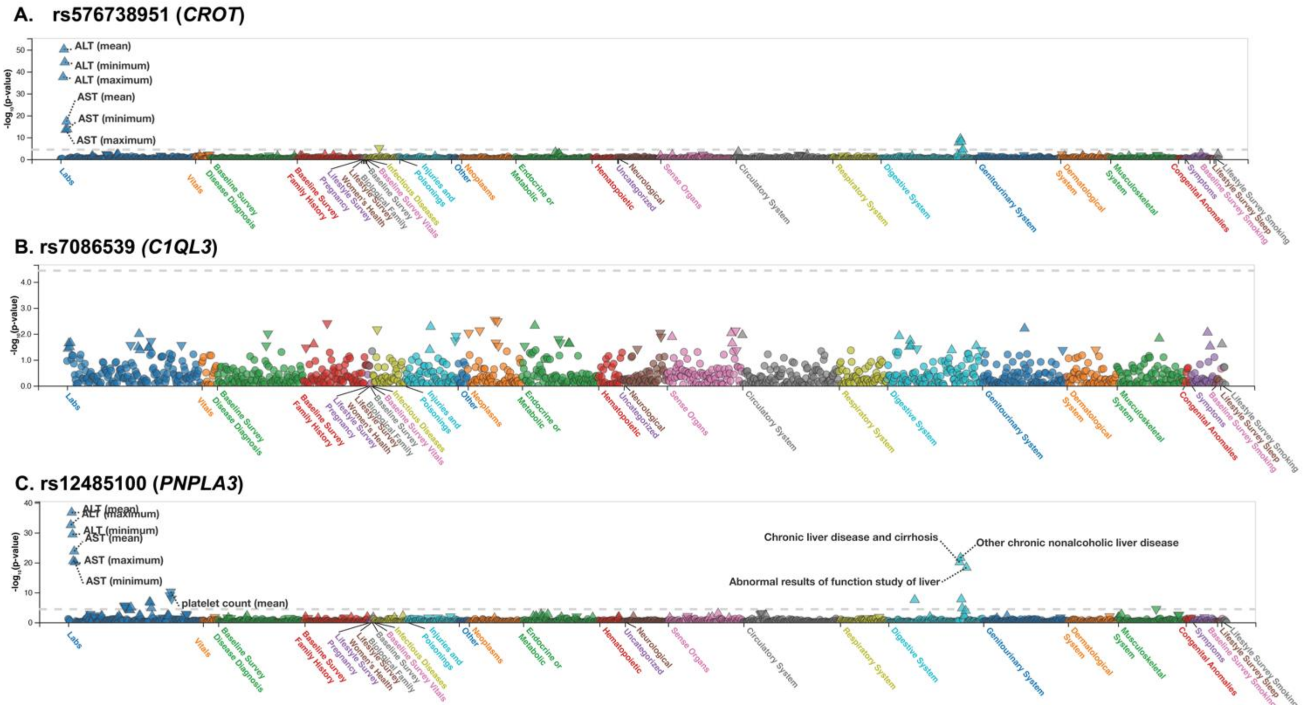

Supplementary Figure 13. PheWAS findings of AST loci variants. Analyses were conducted in the Million Veteran Program. Quantitative traits were inverse-normal transformed. Aspartate transaminase. Rs56331215 and rs71497225 were missing in the database.

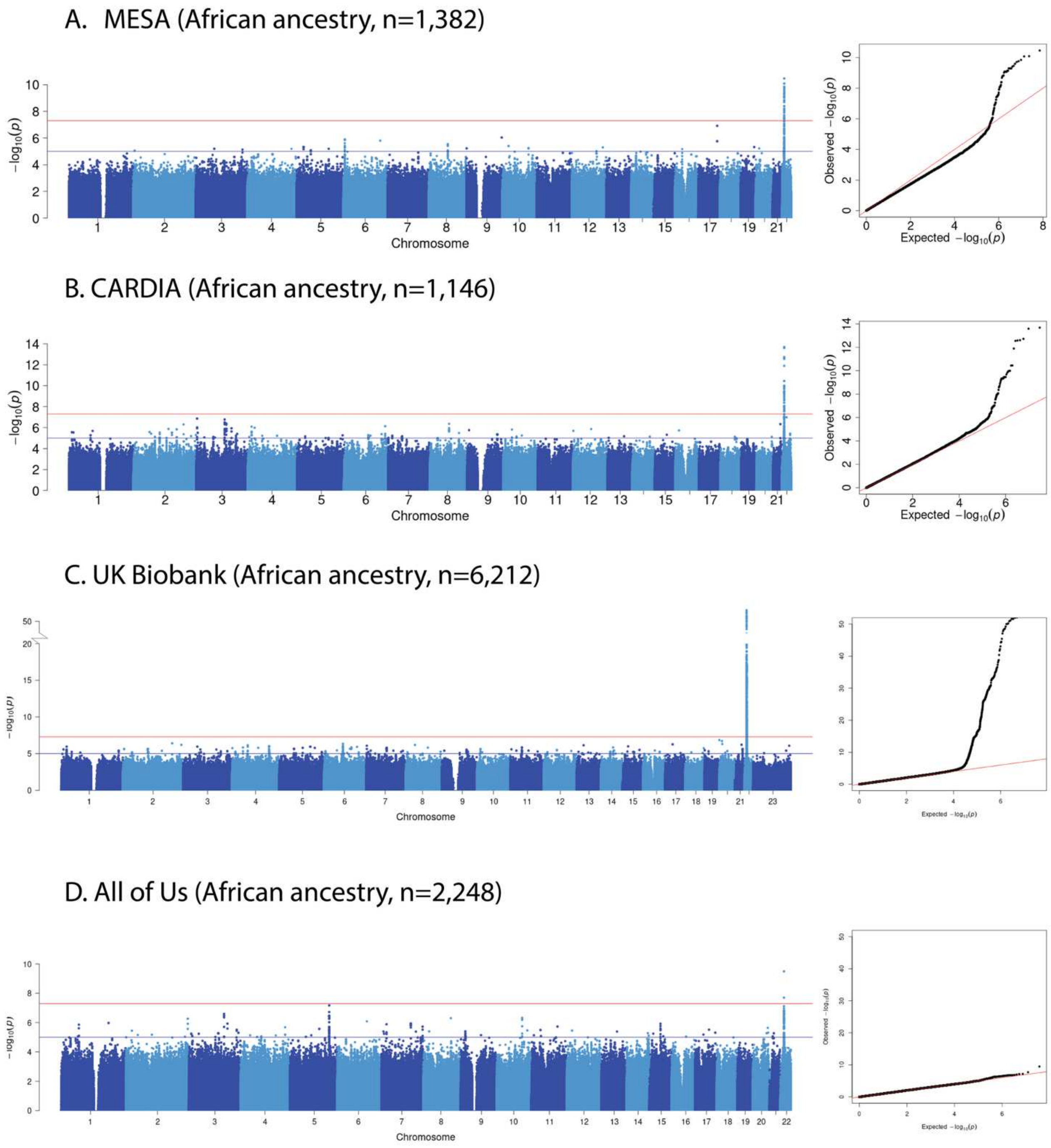

Supplementary Figure 14. Manhattan and quantile-quantile plots of GWAS of gamma-glutamyl transferase levels by cohort.

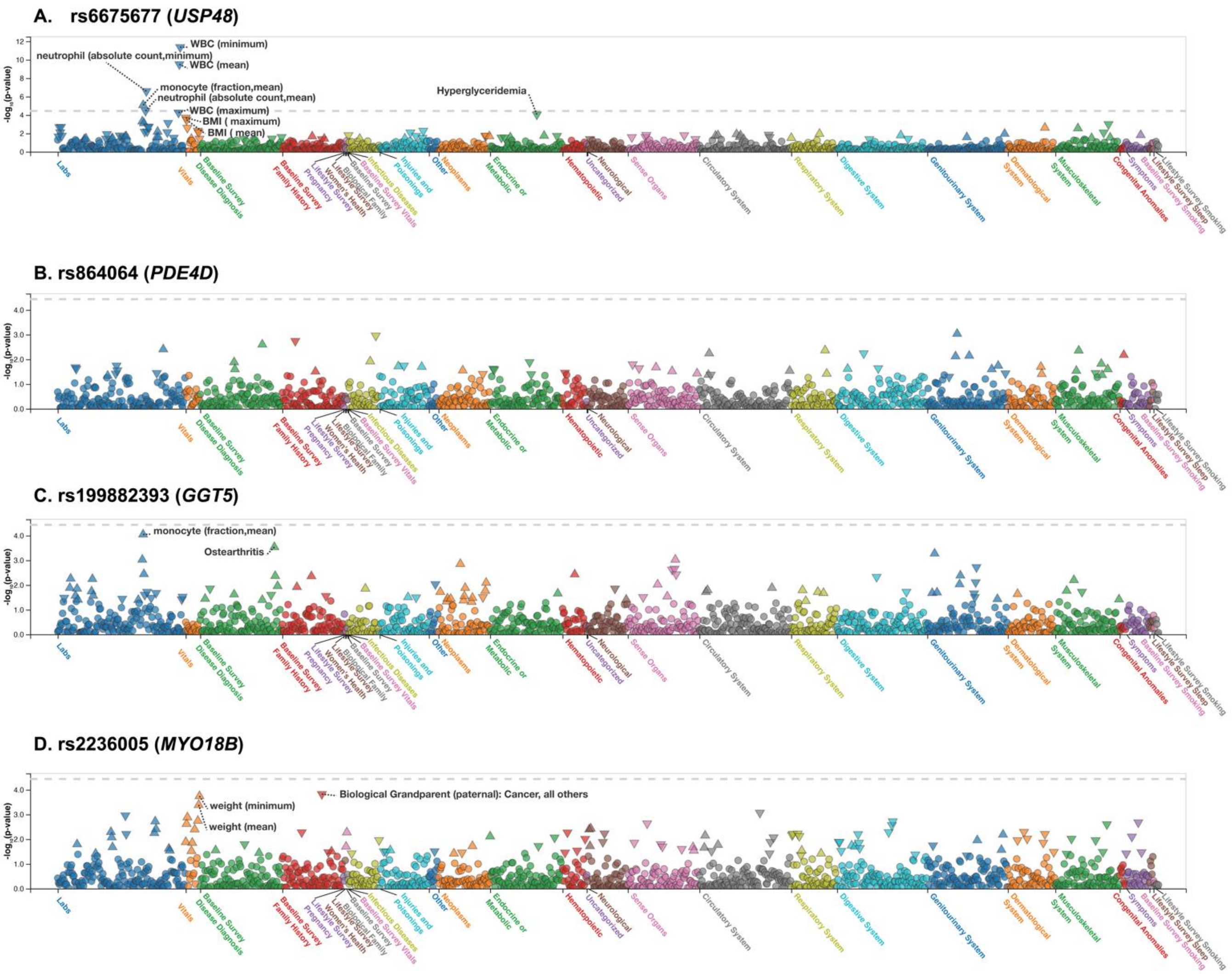

Supplementary Figure 15. PheWAS findings of gamma-glutamyl transferase loci variants . Analyses were conducted in the Million Veteran Program. Quantitative traits were inverse-normal transformed.
